# Piezo1 activation using Yoda1 inhibits macropinocytosis in A431 human epidermoid carcinoma cells

**DOI:** 10.1038/s41598-022-10153-8

**Published:** 2022-04-15

**Authors:** Masashi Kuriyama, Hisaaki Hirose, Toshihiro Masuda, Masachika Shudou, Jan Vincent V. Arafiles, Miki Imanishi, Masashi Maekawa, Yuji Hara, Shiroh Futaki

**Affiliations:** 1 Institute for Chemical Research, Kyoto University, Uji, Kyoto 611-0011 Japan; 2 Division of Analytical Bio-Medicine, Advanced Research Support Center, Ehime University, Toon, Ehime 791-0295 Japan; 3 Division of Cell Growth and Tumor Regulation, Proteo-Science Center, Ehime University, Toon, Ehime 791-0295 Japan; 4 Department of Biochemistry and Molecular Genetics, Ehime University Graduate School of Medicine, Toon, Ehime 791-0295 Japan; 5 Department of Synthetic Chemistry and Biological Chemistry, Graduate School of Engineering, Kyoto University, Katsura, Kyoto 615-8510 Japan; 6 Present Address: Division of Physiological Chemistry and Metabolism, Graduate School of Pharmaceutical Sciences, Keio University, Minato-ku, Tokyo, 105-8512 Japan; 7 Present Address: School of Pharmaceutical Sciences, University of Shizuoka, Shizuoka, 422-8526 Japan

**Keywords:** Chemical tools, Mechanism of action, Membranes

## Abstract

Macropinocytosis is a type of endocytosis accompanied by actin rearrangement-driven membrane deformation, such as lamellipodia formation and membrane ruffling, followed by the formation of large vesicles, macropinosomes. Ras-transformed cancer cells efficiently acquire exogenous amino acids for their survival through macropinocytosis. Thus, inhibition of macropinocytosis is a promising strategy for cancer therapy. To date, few specific agents that inhibit macropinocytosis have been developed. Here, focusing on the mechanosensitive ion channel Piezo1, we found that Yoda1, a Piezo1 agonist, potently inhibits macropinocytosis induced by epidermal growth factor (EGF). The inhibition of ruffle formation by Yoda1 was dependent on the extracellular Ca^2+^ influx through Piezo1 and on the activation of the calcium-activated potassium channel KCa3.1. This suggests that Ca^2+^ ions can regulate EGF-stimulated macropinocytosis. We propose the potential for macropinocytosis inhibition through the regulation of a mechanosensitive channel activity using chemical tools.

## Introduction

Macropinocytosis is a large-scale endocytic pathway that accompanies membrane ruffling driven by actin rearrangement, followed by ruffle closure to form a large vesicle called macropinosome (0.2–10 µm in diameter)^1–3^. Since macropinosomes are significantly larger than vesicles produced by other endocytic pathways (~ 100 nm in diameter), macropinocytosis can non-selectively engulf a large volume of extracellular medium containing amino acids and proteins^3,4^. Pathologically, macropinocytosis plays a pivotal role in promoting the survival of cancer cells through non-selective uptake of extracellular proteins and nutrients^5–7^, suggesting that the development of macropinocytosis inhibitors could be applicable for cancer therapy. From the viewpoint of the intracellular drug delivery, macropinocytosis has been applied and manipulated as an efficient internalization route for the uptake of macromolecules such as antibodies, proteins, and drugs^8,9^. Therefore, finding a new method to manipulate macropinocytosis contributes not only to the development of cancer drugs but also to drug delivery strategies.

Macropinocytosis can be divided into two distinct forms: stimulated macropinocytosis and constitutive macropinocytosis^10^. Growth factors such as epidermal growth factor (EGF) and platelet-derived growth factor (PDGF), chemokines such as stromal cell-derived factor 1α (SDF1α, also known as CXCL12), and chemical compounds such as phorbol 12-myristate 13-acetate (PMA) can induce macropinocytosis in a variety of cells^11–14^. EGF stimulations activate phosphatidylinositol 3-kinase (PI3K) and small GTPases such as Rac1, leading to actin rearrangement and sequential conversion of phosphoinositides, followed by dynamic membrane ruffling, ruffle closure, and formation of macropinosomes^15^. In contrast, macropinocytosis is constitutively-active without a stimulus in macrophages, dendritic cells, and Ras-transformed cancer cells. The constitutive macropinocytosis in macrophages is dependent on calcium-sensing receptors (CaSR) and extracellular calcium ions (Ca^2+^)^16^. However, it remains unclear whether Ca^2+^ affect the regulation of EGF-stimulated macropinocytosis.

In this study, we first investigated the effect of Ca^2+^ influx on macropinocytosis by activation of Ca^2+^ channels using their agonists. We found Yoda1, an agonist of Piezo1, most potently inhibits EGF-stimulated macropinocytosis. Piezo1 is a mechanosensitive Ca^2+^-permeable cation channel^17^. Piezo1 is activated by mechanical stimuli^18^ as well as Yoda1, which was discovered by a high-throughput screening to activate Piezo1^19^. Yoda1 is a known Piezo1-specific agonist and is generally used to activate Piezo1^20^. This work suggests that Yoda1 enhances Ca^2+^ influx followed by aberrant activation of the calcium-activated potassium channel KCa3.1 and inhibition of Rac1 activation. Importantly, we show that Ca^2+^ is a key factor in the regulation of EGF-stimulated macropinocytosis, implying that controlling Ca^2+^ channels such as mechanosensitive channels has the potential to regulate EGF-stimulated macropinocytosis.

## Results

### Ca^2+^ channel activation inhibits EGF-stimulated macropinocytosis in A431 cells

We used the human epidermoid carcinoma cell line A431, which expresses high levels of the EGFR and is a representative cell line for research on EGF-stimulated macropinocytosis. EGF-stimulated macropinocytosis in A431 cells can be evaluated by the amount of cellular uptake of tetramethylrhodamine (TMR)-conjugated dextran 70 kDa (TMR-dex70), a macropinosome marker, using flow cytometry and confocal microscopy analysis^21^.

**Figure 1:**
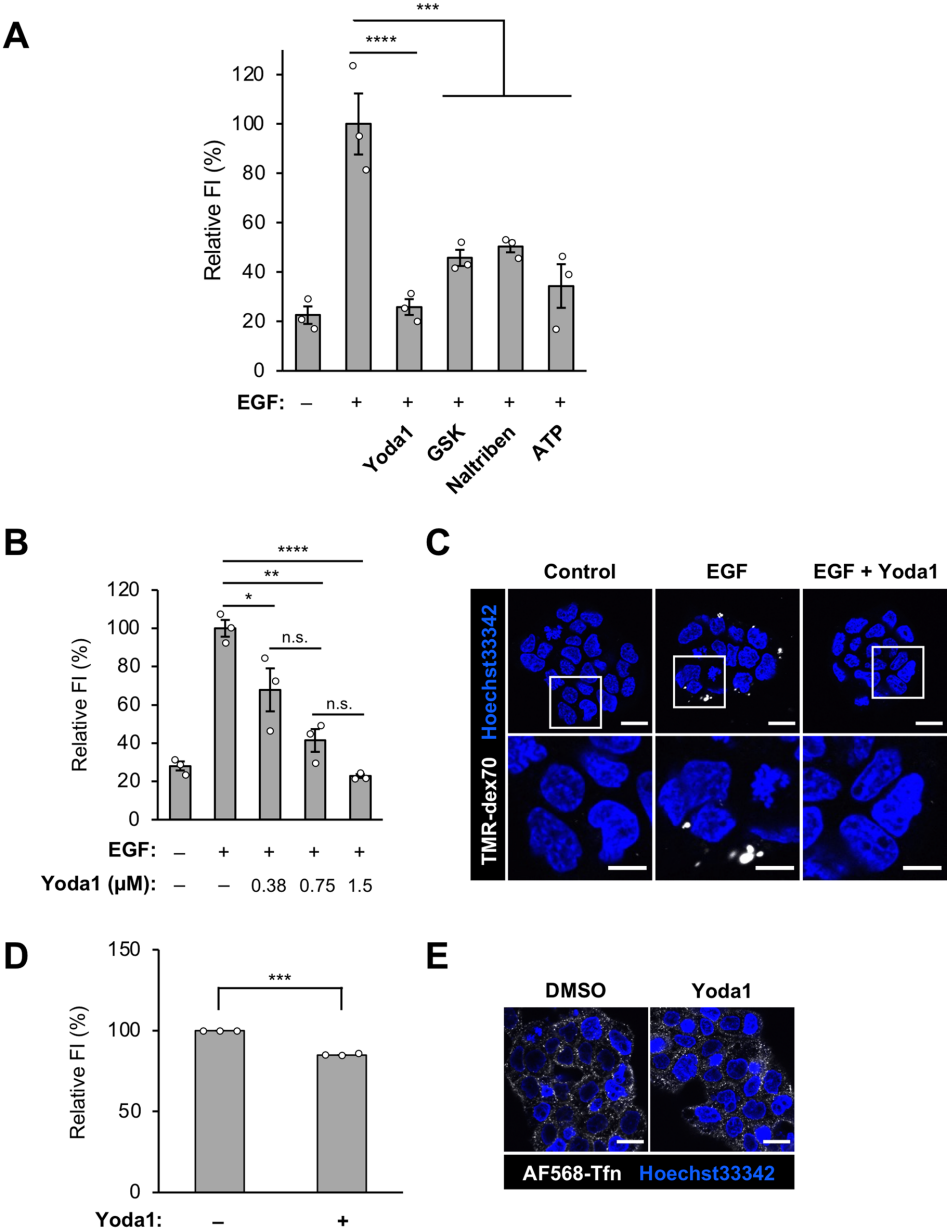
Piezo1 agonist Yoda1 inhibits macropinocytosis induced by EGF in A431 cells. (**A**) Flow cytometry analysis of EGF-stimulated TMR-dex70 uptake with Yoda1 (1.5 µM), GSK (GSK-1016790A, 1 µM), naltriben (50 µM) or ATP (50 µM) for 10 min. (**B**) Flow cytometry analysis of EGF-stimulated TMR-dex70 uptake with the indicated concentration of Yoda1 for 10 min. (**C**) Observation of EGF-stimulated TMR-dex70 uptake into the cells in the absence or presence of Yoda1 (1.5 µM). The bottom images show enlarged views of the areas outlined by the white squares in the top images. (**D**) Flow cytometry analysis of AF568-Tfn uptake into A431 cells. The cells were incubated with AF568-Tfn (20 µg/mL) in the absence or presence of Yoda1 (1.5 µM) for 10 min. (**E**) Confocal microscopy observation of AF568-Tfn uptake. A431 cells were treated as (**D**). Data represent the mean ± s.e.m. (n = 3 independent biological replicates in (**A**), (**B**), and (**D**). *p < 0.05; **p < 0.01; ***p < 0.001; ****p < 0.0001; *n.s.* not significant (one-way ANOVA followed by Tukey–Kramer’s post hoc test (**A**,**B**), Student’s *t* test (D). Scale bars, (C, E) 20 µm; (C bottom) 10 µm.

To assess whether activation of representative Ca^2+^ channels at the plasma membranes, Piezo1, Transient Receptor Potential Vanilloid 4 (TRPV4), Transient Receptor Potential Melastatin 7 (TRPM7), and other receptors such as a variety of P2X receptors and purinergic receptors is involved in the regulation of EGF-stimulated macropinocytosis in A431 cells, we used their agonists, Yoda1, GSK-1016790A (GSK), naltriben, and ATP, respectively. A431 cells were incubated with TMR-dex70 in the presence or absence of EGF and the agonists of Ca^2+^ channels, and cellular uptake of TMR-dex70 was quantitatively evaluated by flow cytometry and observed by confocal microscopy. EGF treatment enhanced the cellular uptake of TMR-dex70, whereas Ca^2+^ channel activation significantly inhibited the EGF-induced uptake of TMR-dex70 (Fig. 1A). These data suggest that any Ca^2+^ source inhibits macropinocytosis. We hypothesized that Ca^2+^ influx might be one such source. For the subsequent experiments to study the effect of Ca^2+^ signaling on macropinocytosis, we chose Yoda1, a highly specific agonist to its target (Piezo1), so that we could exclude side effects from non-specific protein binding. Yoda1 inhibited EGF-induced TMR-dex70 uptake in a concentration-dependent manner, indicating that Yoda1 (1.5 µM) showed the greatest effect (Fig. 1B,C). To check whether Yoda1 specifically affects macropinocytosis, we examined Transferrin (Tfn) uptake, which is internalized by clathrin-mediated endocytosis pathway, in the absence or presence of Yoda1. Yoda1 inhibited only 10% of Alexa Fluor 568-labeled Tfn into A431 cells, indicating that Yoda1 does not substantively affect clathrin-mediated endocytosis (Fig. 1D,E). We performed a cell viability assay (WST-8 assay) to evaluate the cytotoxicity of Yoda1 in A431 cells (Fig. S1). No significant cytotoxicity was observed in starved A431 cells when treated with Yoda1 (1.5 µM) for 30 min (Fig. S1A). Higher concentrations of Yoda1 (3 µM) slightly decreased the cell viability (to ~ 90%) under these conditions. A longer incubation time (4 h) led to significant but weak cytotoxicity (~ 80% cell viability) in starved A431 cells (Fig. S1B). In contrast, the non-starved A431 cells treated with Yoda1 (1.5 µM) for 4 h showed no significant cytotoxicity (Fig. S1C). These results confirm that Yoda1 does not cause cytotoxicity in A431 cells under our experimental conditions (1.5 µM of Yoda1, 10 min). Altogether, these data suggest that activation of Ca^2+^ channels, especially Piezo1, potently inhibits EGF-stimulated macropinocytosis.

### Inhibition of EGF-stimulated macropinocytosis by Yoda1 is dependent on Piezo1

**Figure 2:**
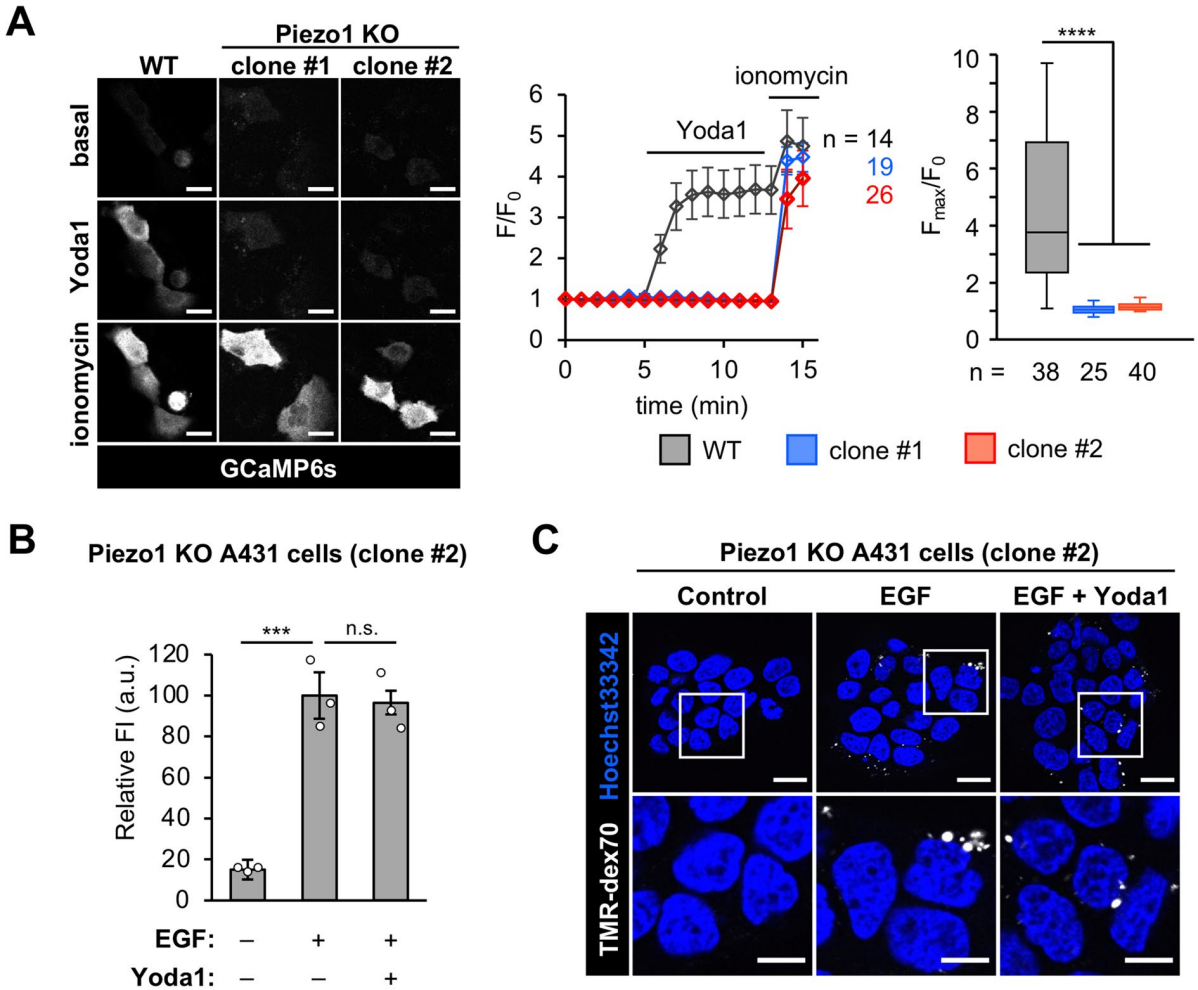
Macropinocytosis inhibition by Yoda1 is Piezo1-dependent. (**A**) GCaMP6s fluorescence intensities in WT, Piezo1-KO clone#1 and clone #2 cells were recorded every 1 min. Yoda1 (1.5 µM) and ionomycin (5 µM) were added at 5 and 13 min after start of time-lapse imaging, respectively. Left: Representative images of GCaMP6s-expressing cells before and after addition of Yoda1 and ionomycin. Middle: Representative time-course of relative fluorescence intensity of GCaMP6s. Right: Quantification of maximum Yoda1-induced GCaMP6s intensity increase. Box and whiskers graph: line, median; box, upper and lower quartiles; whiskers, maxima and minima. (**B**) Flow cytometry analysis of EGF-stimulated TMR-dex70 uptake into Piezo1-KO A431 cells (clone #2) in the absence or presence of Yoda1 (1.5 µM) for 10 min. (**C**) Observation of EGF-stimulated TMR-dex70 uptake into Piezo1-KO A431 cells treated as (**B**). The bottom images show enlarged views of the areas outlined by the white squares in the top images. Data represent the mean ± s.e.m. (n = 3 independent biological replicates in (**B**); n = 14, 19, 26 cells for WT, clone #1 and #2, respectively) in (**A**, middle). Data represent in box plot (from left to right, n = 38, 25, 40 cells pooled from two independent experiments) in (**A**, right). ***p < 0.001; ****p < 0.0001; *n.s.* not significant [one-way ANOVA followed by Dunnett’s post hoc test (**A**) or one-way ANOVA followed by Tukey–Kramer’s post hoc test (**B**)]. Scale bars, (**C**, top) 20 µm; (**C**, bottom) 10 µm.

To determine whether the inhibitory effect of Yoda1 on macropinocytosis is Piezo1-dependent, we established Piezo1 knockout (KO) A431 cells, using the CRISPR/Cas9 system^22^. We obtained two KO clones (clones #1 and #2) and monitored intracellular Ca^2+^ increase by fluorescence of a genetically encoded Ca^2+^ indicator GCaMP6s^23^. We found that Yoda1-induced Ca^2+^ signaling was completely abolished in these two KO clones (Fig. 2A). We also confirmed that ionomycin, a Ca^2+^ ionophore, induced Ca^2+^ influx in both wild-type (WT) and Piezo1-KO cells. These results indicate that Yoda1-induced Ca^2+^ influx into A431 cells is dependent on Piezo1 (Fig. 2A). We also found that there were the alleles without frameshift mutations (i.e. 33 and 9-bp deletions, resulting in 11 and 3-amino acid deletions of Piezo1 protein, referred to as Δ946–956 and Δ944–946, respectively) in both clones. We further checked whether these deletion-mutants lost the function of Piezo1 by expressing the mutants in HEK293T cells (Fig. S2A and S2B), and then decided to use clone #2 as Piezo1-KO A431 cells for the subsequent experiments. Piezo1 gene expression in A431 WT and Piezo1-KO cells was further confirmed by real-time quantitative PCR (qPCR) (Fig. S2C). The results indicated that the mRNA level of the Piezo1 coding region was decreased by over 90% in the Piezo1-KO cell line, suggesting that there is minimal expression of the Piezo1 mutant in the Piezo1-KO cells. Using the Piezo1-KO A431 cells, we conducted a dextran uptake assay. Flow cytometry analysis and confocal microscopy observation revealed that EGF induced macropinocytosis in Piezo1-KO A431 cells in both the absence and presence of Yoda1 (Fig. 2B,C). These results clearly show that the Piezo1 agonist Yoda1 inhibits macropinocytosis through Piezo1. In addition, compared to the wild-type, the dextran uptake was increased by ~ 30% in Piezo1-KO A431 cells upon EGF-stimulated macropinocytosis (Fig. S2D), implying that Piezo1 might be activated, which in turn, negatively regulates the process.

### Activated Piezo1 inhibits peripheral ruffle formation by blocking Rac1 activation

**Figure 3:**
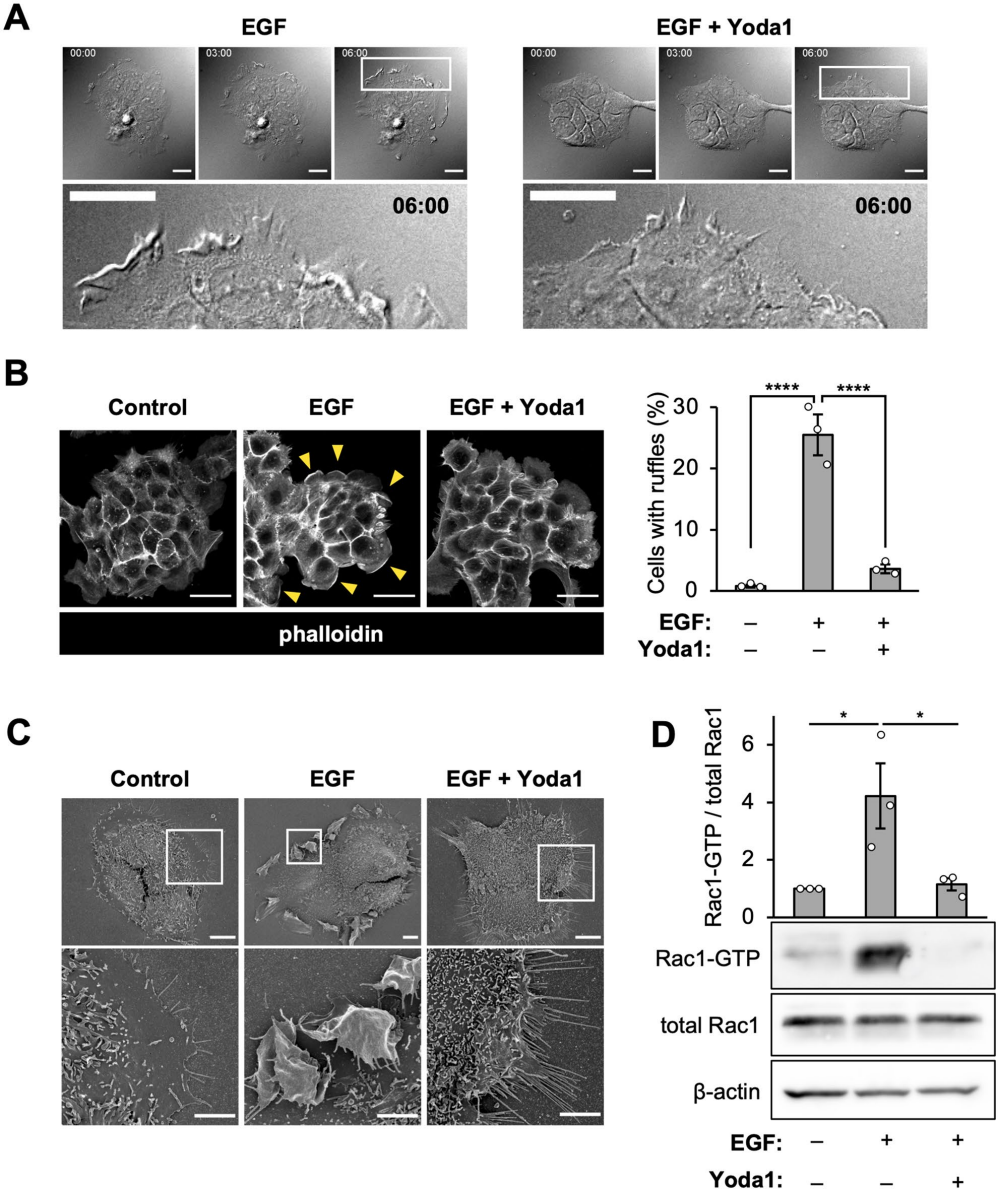
Piezo1 activation inhibits peripheral ruffle formation. (**A**) Live cell imaging of EGF-induced membrane ruffles in A431 cells. The cells were stimulated with EGF in the absence or presence of Yoda1 (1.5 µM). Live cell imaging was started immediately after adding EGF and Yoda1. DIC images at the indicated time points (0, 3 and 6 min) are shown. The bottom images show enlarged views of the areas outlined by the white squares in the images at 6 min. (**B**) F-actin staining with phalloidin. Left: Representative images are shown. Yellow arrowheads indicate peripheral membrane ruffling area. Right: Quantification of cells with peripheral ruffles. Data represent the mean ± s.e.m. (number of total counted cells pooled from three independent experiments: from left to right, 505, 477, and 501). (**C**) Scanning electron microscopy images of A431 cells stimulated with EGF in the absence or presence of Yoda1 (1.5 µM) for 5 min. The bottom images show enlarged views of the areas outlined by the white squares in the top images. (**D**) Rac1 activation pull-down assay. Representative western blot images and densitometric analysis of Rac1-GTP levels normalized to total Rac1 are shown (three independent experiments). ****p < 0.0001 [one-way ANOVA followed by Tukey–Kramer’s post hoc test (**B**,**D**)]. Scale bars, (**A**) 20 µm; (**B**) 50 µm; (**C**, top) 10 µm; (**C**, bottom) 5 µm.

Macropinocytosis is an actin-driven, non-specific endocytosis process accompanied by the following steps: (1) formation of membrane ruffles induced by actin reorganization and (2) subsequent closure of the ruffles to form macropinosomes^24^. To determine which step Yoda1 inhibits, we investigated peripheral ruffle formation by time-lapse live cell imaging and F-actin staining using phalloidin. Peripheral ruffles are actin-rich and sheet-like protrusions of the membrane^25^. In the absence of Yoda1, the extension and folding back of the plasma membrane of A431 cells after EGF addition were clearly observed within 6 min. However, this phenomenon was absent in the presence of Yoda1 (Fig. 3A, Movies 1 and 2). In addition, staining actin filaments using phalloidin revealed that Yoda1 inhibited actin polymerization. A431 cells were stimulated with EGF for 5 min in the presence or absence of Yoda1 and then fixed, followed by staining with rhodamine-phalloidin to detect F-actin; then, the cells with the F-actin positive peripheral ruffles were quantified (Fig. 3B). EGF stimulation resulted in ~ 25% of cells with peripheral ruffles, whereas co-treatment with Yoda1 significantly decreased the proportion of cells with peripheral ruffles (~ 4%). Moreover, scanning electron microscopy clearly showed that Yoda1 inhibited EGF-induced peripheral ruffle formation (Fig. 3C).

We then investigated whether Yoda1 inhibited Rac1 activation. In the process of actin rearrangement to form membrane ruffles, EGF-induced actin rearrangement is due to the activation of the small GTPase Rac1^26^. The pulldown experiment of active Rac1 (Rac1-GTP) showed that the amount of Rac1-GTP in the cells treated with EGF increased as previously reported^27^. However, Yoda1 inhibited EGF-induced increase in the amount of Rac1-GTP (Fig. 3D and Fig. S3). These results show that Yoda1 inhibits Rac1 activation. We confirmed that Yoda1 had almost same inhibitory effect with a Rac1 inhibitor, NSC23766 (Fig. S4). The TMR-dex70 uptake assay showed that NSC23766 (100 µM) also inhibited macropinocytosis. The result also supports that Rac1 activation is critical for EGF-stimulated macropinocytosis and that Yoda1 potently inhibits macropinocytosis.

We next examined three conventional possibilities about inhibition of Rac1. First, we investigated whether Yoda1 lowers cytosolic pH. It has been previously shown that macropinocytosis inhibition by amiloride, an inhibitor of Na^+^/H^+^ exchangers (NHE), is due to lower submembranous pH, which prevents Rac1 activation^27^. We used the dual-emission ratio (645/585 nm) of seminaphthorhodafluor dye-5 (SNARF-5F) to quantify intracellular pH (Fig. S5A)^27^. We then compared the cytosolic pH when A431 cells were treated either with dimethyl sulfoxide (DMSO) as vehicle, Yoda1, or ethyl-isopropyl amiloride (EIPA), an amiloride derivative that is widely used as a macropinocytosis inhibitor^28^. EIPA significantly decreased in cytosolic pH, whereas Yoda1 did not lower cytosolic pH (Fig. S5B), suggesting that Rac1 inhibition by Yoda1 is unlikely due to a decrease in intracellular pH. Second, we examined the possibility of inhibition of EGF signal transduction leading to Rac1 activation. Yoda1 did not inhibit EGF-related signaling pathways such as phosphorylation of EGFR and Vav2 (Fig. S6A, S6B), suggesting that Yoda1 does not affect the acute response of phosphorylation induced by EGF signaling. Finally, we also checked whether Yoda1 affects cholesterol (Chol) distribution in cells. Because membrane ruffling and macropinocytosis in A431 cells require cholesterol to regulate the localization of Rac1^29^ and because Piezo1 activity is also modified by cholesterol^30^, we investigated Chol distribution in the absence or presence of Yoda1 using a genetically encoded biosensor for Chol (mCherry-D4H)^31^. Time-lapse imaging showed that Yoda1 does not affect Chol distribution in the cells, suggesting that inhibition of macropinocytosis by using Yoda1 is not due to change in Chol localization (Fig. S7). Although a further study will be needed to elucidate a mechanism of preventing Rac1 activation, our results suggest that Yoda1 may inhibit Rac1 activation in a different way than previously reported.

### Extracellular Ca^2+^ is required for macropinocytosis inhibition by Piezo1 activation

**Figure 4:**
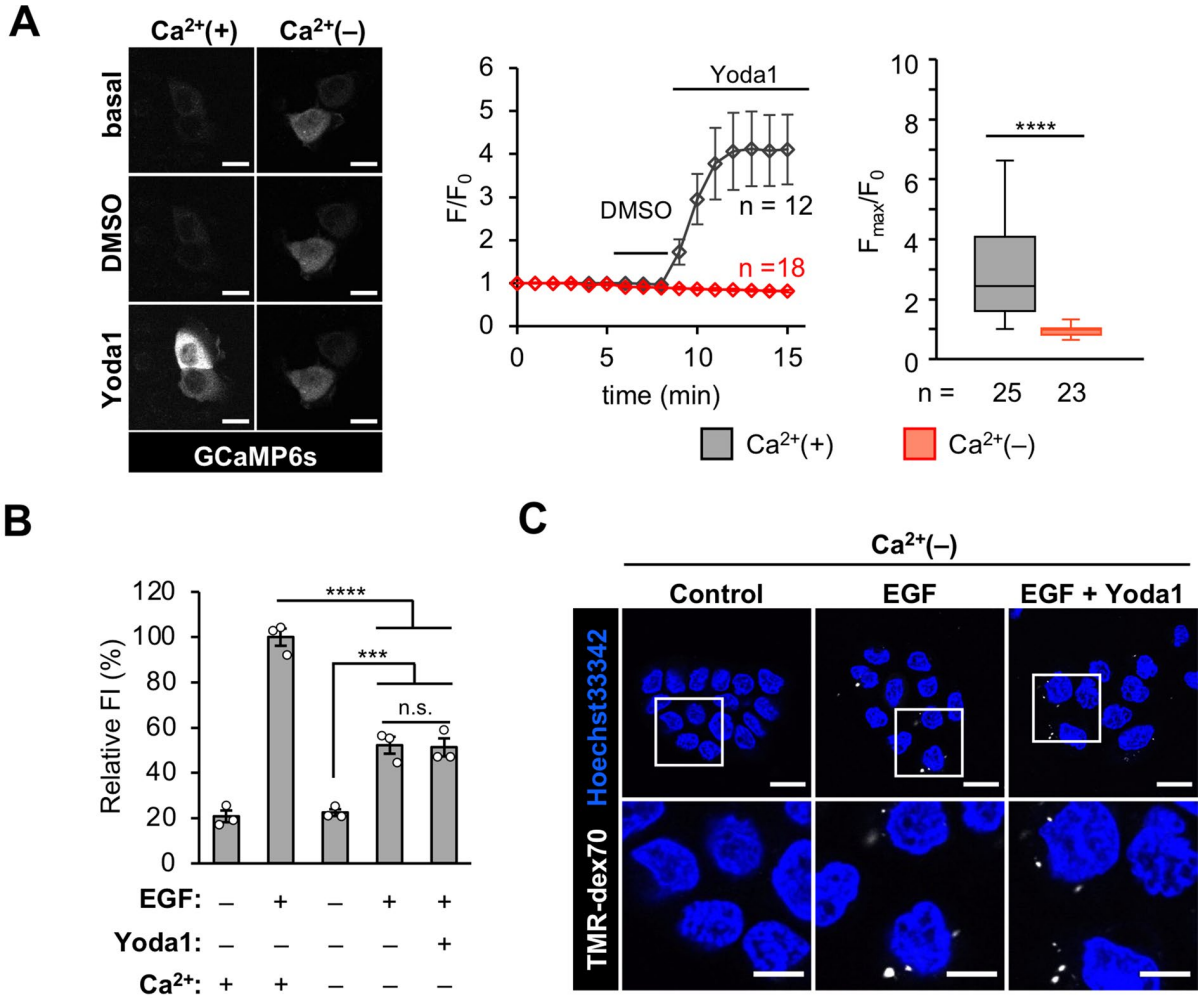
Macropinocytosis inhibition by Yoda1 requires extracellular Ca^2+^ influx. (**A**) GCaMP6s fluorescence intensity was recorded every 1 min. DMSO and Yoda1 (1.5 µM) were added at 5 and 8 min after start of time-lapse imaging, respectively. Left: Representative images of the GCaMP6s-expressing cells treated DMSO and Yoda1 in the absence or presence of Ca^2+^ in culture media. Middle: Representative time-course of relative fluorescence intensity of GCaMP6s. Data represent the mean ± s.e.m. (n = 12 and 18 cells for Ca^2+^(+) and Ca^2+^(−), respectively). Right: Quantification of maximum Yoda1-induced GCaMP6s intensity increase. Data represent in box plot (n = 25 and 23 cells, for Ca^2+^(+) and Ca^2+^(−), respectively, pooled from two independent experiments). Box and whiskers graph: line, median; box, upper and lower quartiles; whiskers, maxima and minima. (**B**) Flow cytometry analysis of EGF-stimulated TMR-dex70 uptake in Ca^2+^-containing or Ca^2+^-free condition in the absence or presence of Yoda1 (1.5 µM). Data represent the mean ± s.e.m. (n = 3 independent biological replicates). (**C**) Observation of TMR-dex70 uptake in Ca^2+^-free condition. The cells were treated as (**B**). The bottom images show enlarged views of the areas outlined by the white squares in the top images. ***p < 0.001; ****p < 0.0001; *n.s.* not significant [Student’s *t* test (**A**) or one-way ANOVA followed by Tukey–Kramer’s post hoc test (**B**)]. Scale bars, (**A**,**C**, top) 20 µm; (**C**, bottom) 10 µm.

Since activated Piezo1 is known to be permeable to extracellular Ca^2+^ influx, we next examined whether extracellular Ca^2+^ influx is important for the inhibition of macropinocytosis by Piezo1 activation using Yoda1. To confirm the effects of Yoda1 on Ca^2+^ influx into A431 cells, we conducted time-lapse calcium imaging using A431 cells transiently expressing GCaMP6s. Yoda1 was added 8 min after time-lapse imaging started, resulting in an immediate increase in intracellular Ca^2+^ concentration (Fig. 4A). Because Piezo1 and other mechanosensitive ion channels could be activated by shear stress such as stimulus by addition of buffer solution itself^32,33^, Hanks’ balanced salt solution (HBSS) containing DMSO was used as a vehicle control. After adding DMSO solution, the fluorescence of GCaMP6s did not increase, as shown in Fig. 4A, indicating that the intracellular Ca^2+^ concentration did not significantly increase. To investigate whether the intracellular calcium response induced by Yoda1 is due to extracellular Ca^2+^ influx, we used Ca^2+^-free HBSS. Under these conditions, Yoda1 did not increase intracellular Ca^2+^ concentrations (Fig. 4A). These results indicate that extracellular Ca^2+^ influx is crucial for the increase in intracellular Ca^2+^ concentrations caused by the addition of Yoda1.

We then investigated whether Yoda1 inhibits macropinocytosis also in Ca^2+^-free conditions. The dextran uptake assay was conducted using a Ca^2+^-free medium. Yoda1 did not inhibit TMR-dex70 uptake in the absence of extracellular Ca^2+^, indicating that extracellular Ca^2+^ influx through Piezo1 is crucial for the inhibition of macropinocytosis by Yoda1 (Fig. 4B,C). EGF-induced uptake of TMR-dex70 in Ca^2+^-free medium without Yoda1 was significantly reduced (by ~ 40%) compared to that in Ca^2+^-containing medium. EGF-stimulated macropinocytosis in A431 cells has been previously reported to be independent of extracellular Ca^2+^ ion^16^, but the effect of extracellular Ca^2+^ may vary, likely due to differences in experimental conditions and assay systems. This result indicates that Yoda1 did not inhibit EGF-stimulated macropinocytosis under extracellular Ca^2+^-free conditions. These data suggest that Piezo1 activation by Yoda1 inhibits macropinocytosis in an extracellular Ca^2+^-dependent manner.

### KCa3.1 activation is necessary for the inhibitory effect of Yoda1 on ruffle formation

We then sought to identify molecule(s) that function downstream of Yoda1-induced Ca^2+^ signaling related to macropinocytosis inhibition. We focused on KCa3.1, a Ca^2+^-activated K^+^ channel that is activated by Ca^2+^ influx through Piezo1 and reduce cell volume in red blood cells^34^. KCa3.1 also plays a key role in EGF-stimulated micropinocytosis^21^. In macropinocytosis, sequential dephosphorylation of phosphoinositides (PI(3,4,5)P_3_ → PI(3,4)P_2_ → PI(3)P → PI) is required^35^. KCa3.1 has been reported to be activated by PI(3)P and is also crucial for macropinocytic cup formation^21,36^. Therefore, proper temporal activation of KCa3.1 at a later stage of the macropinocytosis process is required for completion of macropincytosis.

**Figure 5:**
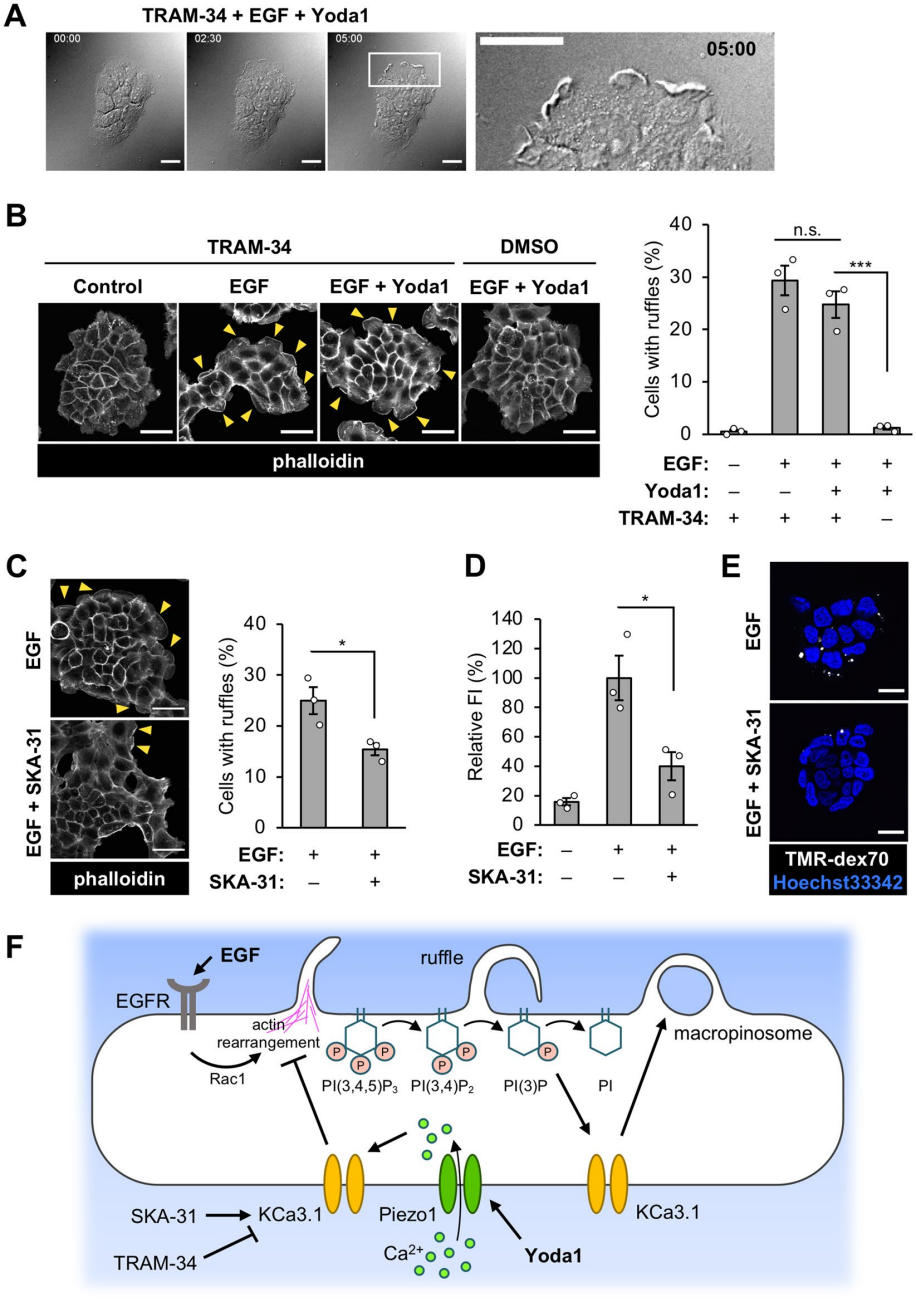
Involvement of KCa3.1 in inhibitory effect of Yoda1 on ruffle formation. (**A**) Live cell imaging of EGF-induced membrane ruffles in A431 cells. The cells were pretreated with a KCa3.1 inhibitor TRAM-34 for 5 min and then stimulated with EGF in the presence of Yoda1 (1.5 µM). Live cell imaging was started immediately after adding EGF and Yoda1. DIC images at the indicated time points (0, 2.5 and 5 min) are shown. The right image shows an enlarged view of the area outlined by the white square in the image at 5 min. (**B**) F-actin staining with phalloidin. Left: Representative images of A431 cells pretreated with TRAM-34 for 5 min and then stimulated with EGF in the absence or presence of Yoda1 (1.5 µM) for 5 min. Right: Quantification of cells with peripheral ruffles. (**C**) F-actin staining with phalloidin. Left: Representative images of A431 cells pretreated with KCa3.1 activator SKA-31 for 5 min and then stimulated with EGF for 5 min. Right: Quantification of cells with peripheral ruffles. (**D**) Flow cytometry analysis of TMR-dex70 uptake. The cells were pretreated with KCa3.1 activator SKA-31 for 5 min and then stimulated with EGF for the uptake of TMR-dex70 for 10 min. (**E**) Observation of TMR-dex70 uptake. The cells were treated as in (**D**). (**F**) Working hypothesis of macropinocytosis inhibition using Yoda1. Yoda1-induced, Piezo1-dependent extracellular Ca^2+^ influx causes non-proper activation of KCa3.1, which inhibits Rac1 activation followed by ruffle formation. PowerPoint (version 16.57, Microsoft) was used to generate the picture. Data in (**B**) and (**C**) represent the mean ± s.e.m. (number of total counted cells pooled from three independent experiments: from left to right, (**B**) 540, 559, 562, and 552; (**C**) 538 and 561). Data in (**D**) represent the mean ± s.e.m. (n = 3 independent biological replicates). *p < 0.05; ***p < 0.001; *n.s.* not significant [one-way ANOVA followed by Tukey–Kramer’s post hoc test (**B**,**D**) or Student’s *t* test (**C**)]. Yellow arrowheads in (**B**) and (**C**) indicate F-actin positive peripheral membrane ruffling area. Scale bars, (**A**,**E**) 20 µm; (**B**,**C**) 50 µm.

We hypothesize that Yoda1-induced Ca^2+^ influx acutely activates KCa3.1, and that the improper activation of KCa3.1 inhibits ruffle formation. Since inhibition of KCa3.1 impairs macropinosome formation but does not affect ruffle formation^21^, the involvement of KCa3.1 in the inhibition of macropinocytosis by Yoda1 was tested by a membrane ruffling assay. A431 cells were pretreated with TRAM-34, a potent and selective KCa3.1 inhibitor, and then treated with EGF and Yoda1 in the presence of TRAM-34. Live cell differential interference contrast (DIC) imaging and phalloidin staining showed that KCa3.1 inhibition by TRAM-34 restored EGF-induced peripheral ruffle formation in the presence of Yoda1 (yellow arrowheads, Fig. 5A,B and Movie 3). We also compared the effects of Yoda1 with ionomycin on the inhibition of macropinocytosis. Ionomycin treatment led to increased intracellular Ca^2+^ concentration (Fig. 2A), and it has been reported that ionomycin induces phospholipase C (PLC) activation to hydrolyze PI(4,5)P_2_ into diacylglycerol (DAG) and inositol-3-phosphate (IP_3_)^37^. PI(4,5)﻿P_2_ breakdown is thought to lead to inhibition of macropinocytosis, because sequential phosphorylation and dephosphorylation of PI(4,5)﻿P_2_ is required. Therefore, we checked the amount of PI(4,5)P_2_ in the plasma membrane using a genetically encoded biosensor of PI(4,5)P_2_ (GFP-PLCδ-PH)^38^. Time-lapse imaging showed that ionomycin treatment led to complete redistribution of the PI(4,5)﻿P_2_ biosensor on the plasma membrane to the cytosol as previously reported^37^, whereas Yoda1 treatment did not induce the redistribution of the biosensor (Fig. S8A). This suggests that Yoda1 treatment, unlike ionomycin, does not hydrolyze PI(4,5)﻿P_2_ despite increased intracellular Ca^2+^ concentration. In addition, A431 cells pretreated with TRAM-34 did not recover peripheral membrane ruffling in the presence of ionomycin during EGF stimulation (Fig. S8B and Movie 4). Altogether, these results suggest that the inhibitory effect of Yoda1 on EGF-stimulated macropinocytosis is related to KCa3.1 activation by an increase in Ca^2+^ concentration through Piezo1 activation; however, the mechanism by which Yoda1 inhibits macropinocytosis is different from that of ionomycin.

### Pharmacological activation of KCa3.1 inhibits EGF-induced macropinocytosis

To further confirm that KCa3.1 activation is involved in the inhibition of macropinocytosis, we next investigated whether the pharmacological activation of KCa3.1 by using SKA-31, a potent potassium channel activator, inhibits EGF-induced membrane ruffle formation and macropinocytic uptake. SKA-31 is known to activate KCa3.1 and KCa2^39^. A431 cells were stimulated with EGF in the absence or presence of SKA-31, and a membrane ruffling assay was performed (Fig. 5C). Although SKA-31 did not completely inhibit EGF-mediated membrane ruffle induction compared to Yoda1, there was a significant decrease in the formation of membrane ruffles induced by EGF. Inhibition of macropinocytosis by SKA-31 was also confirmed by flow cytometry analysis and confocal microscopy observation of TMR-dex70 uptake (Fig. 5D,E). SKA-31 significantly inhibited the EGF-dependent uptake of TMR-dex70. Altogether, these results indicate that KCa3.1, if activated in a non-temporal manner, can inhibit EGF-stimulated macropinocytosis.

Taken together with our findings in this study, it is suggested that Yoda1 specifically activates Piezo1, which leads to acute activation of KCa3.1, followed by inhibition of actin rearrangement due to preventing Rac1 activation (Fig. 5F).

## Discussion

Macropinocytosis has recently attracted much more attention, especially from the point of view of cancer metabolism^5,40,41^. Since macropinocytosis functions as a nutrient supply pathway in cancer cells, preventing macropinocytosis in cancer cells is thought to be an important method for cancer therapy^42^. Therefore, further understanding of the molecular mechanisms and physiological significance of macropinocytosis is required. Unfortunately, there are very few specific and versatile inhibitors for macropinocytosis, because few specific proteins and lipids related to macropinocytosis have been identified^28^. This makes it challenging to develop specific pharmacological tools to inhibit macropinocytosis^43^. Conventional macropinocytosis inhibitors such as cytochalasin D, wortmannin, and EIPA can affect other endocytic pathways or have off-target effects. For instance, cytochalasin D not only blocks macropinocytosis but affects receptor-mediated endocytosis^44^. Wortmannin blocks membrane ruffle closure of macropinocytosis by inhibiting PI3K^45^. Although wortmannin is considered to be a highly selective inhibitor of PI3K, it can also potently inhibit mammalian polo-like kinase 1 (PLK1), which is critical in mitosis^46^. EIPA is one of the most common reagents to inhibit macropinocytosis by blocking NHE, but it also blocks transient receptor potential polycystic 3 (TRPP3), a Ca^2+^-activated channel belonging to the TRP superfamily of cation channels^47^. By contrast, Yoda1 is a specific agonist of Piezo1 and inhibited macropinocytosis more specifically than clathrin-mediated endocytosis (Fig. 1), suggesting that Yoda1 is a chemical tool as a selective potent inhibitor of macropinocytosis.

Our study implies that Piezo1 might be involved in the negative regulation of macropinocytosis to some extent. To investigate the Yoda1-induced inhibition of macropinocytosis, we established Piezo1-KO cell lines. Our study showed that these cells could perform EGF-stimulated macropinocytosis and that Yoda1 had no effect on the process in Piezo1-KO cells (Fig. 2). Interestingly, Piezo1-KO cells increased the amount of TMR-dex70 uptake by EGF-stimulated macropinocytosis compared to the WT A431 cells (Fig. S2D). Our study suggests that Piezo1 activation leads to macropinocytosis inhibition. Therefore, higher cellular TMR-dex70 uptake into Piezo1-KO cells might imply that Piezo1 negatively regulates macropinocytosis. It has been reported that the cytoskeleton and the membrane lipids such as phosphoinositides are involved in Piezo1 activation^48–50^. Considering that the process of macropinocytosis accompanies phosphoinositide conversion and dynamic actin rearrangement, Piezo1 might be activated in the region where macropinocytosis occurs. However, further studies are required to elucidate the physiological role of Piezo1 in macropinocytosis regulation.

In this study, we showed that Yoda1 treatment led to inhibition of Rac1 activation, which inhibited peripheral membrane ruffle formation (Fig. 3). It was reported that knockdown of Piezo1 in gastric cancer cells led to Rac1 activation^51^. This previous study may suggest that Piezo1 activation inhibits Rac1, although the mechanism still remains unclear. Importantly, we further showed that the inhibitory effect of Yoda1 on macropinocytosis was dependent on extracellular Ca^2+^ influx through Piezo1 (Fig. 4). The inhibition of KCa3.1, which is a calcium-activated potassium channel, recovered the EGF-stimulated membrane ruffle formation (i.e. actin rearrangement) even in the presence of Yoda1 (Fig. 5A,B). This suggests that EGF-stimulated actin rearrangement can be induced even in the presence of Yoda1 on condition that KCa3.1 is inhibited. Therefore, we propose that Piezo1 activation followed by KCa3.1 activation likely leads to the inhibition of actin rearrangement. A previous study reported that KCa3.1 activation is essential in membrane ruffle closure, the later stage of macropinocytosis process^21^. On the other hand, we showed that a KCa3.1 activator as well as Yoda1 also impaired macropinocytosis (Fig. 5C–E). Altogether, our results suggest that appropriate temporal activation of KCa3.1 is important in macropinocytosis, and that KCa3.1 activation, following acute Ca^2+^ influx induced by Yoda1, could lead to the inhibition of actin rearrangement (Fig. 5F).

In conclusion, this work is the first to show that the activation of Piezo1 using Yoda1 potently inhibits EGF-stimulated macropinocytosis in A431 cells. Moreover, our results showed that extracellular Ca^2+^ influx through Piezo1 modulates EGF-stimulated macropinocytosis, suggesting the impact of Ca^2+^ on the regulation of EGF-stimulated macropinocytosis. This study paves the way for the development of methods to manipulate macropinocytosis by regulating Ca^2+^ channel activity using chemical tools.

## Materials and methods

### Reagents

Reagents and antibodies used in this study are listed in Table S1 and Table S2, respectively. Each reagent was dissolved in the recommended solvent, aliquoted, and stored at − 30 °C. Other reagents and culture media were also purchased either from FUJIFILM Wako Pure Chemical Corporation, Sigma-Aldrich, or Thermo Fisher Scientific, unless otherwise specified.

### Cell culture

Cell lines used in this study are listed in Table S3. A431 cells were cultured in Dulbecco’s modified Eagle’s medium (D-MEM, high glucose) (FUJIFILM Wako Pure Chemical Corporation) supplemented with 10% (v/v) heat-inactivated fetal bovine serum (FBS) (Gibco) [D-MEM(+)]. HEK293T cells were cultured in D-MEM with low glucose (FUJIFILM Wako Pure Chemical Corporation) supplemented with 10% (v/v) heat-inactivated FBS. All cells were maintained at 37 °C in a humidified 5% CO_2_ incubator and passaged every 2–4 days. Cells were used for experiments between passage numbers 1 and 15.

### Plasmids construction

Plasmids and primers used in this study are listed in Table S4 and Table S5, respectively. pGP-CMV-GCaMP6s-CAAX was a gift from Dr. Tobias Meyer^52^. pGP-CMV-GCaMP6s was generated by deleting the CAAX sequence from pGP-CMV-GCaMP6s-CAAX. To construct the expression plasmid pIRES2-mCherry, cDNA encoding ZsGreen1 was removed from pIRES2-ZsGreen1 (Takara) by digestion with BstXI/NotI, and then an mCherry cDNA fragment was inserted into the same sites. pPiezo1-IRES2-mCherry was generated by inserting human Piezo1 cDNA into the EcoRI/BamHI sites of pIRES2-mCherry. pPiezo1(Δ946–956)-IRES2-mCherry and pPiezo1(Δ944–946)-IRES2-mCherry were generated by switching from the WT to the deleted sequences between the MluI and SalI sites of pPiezo1-IRES2-mCherry. The deleted sequences were generated as follows: two sequences for each deleted sequence were amplified from pPiezo1-IRES2-mCherry using the following pairs of primers: Piezo1 mutant forward 1 and Piezo1 mutant reverse1, Piezo1 mutant forward2 and Piezo1 mutant reverse2, Piezo1 mutant forward1 and Piezo1 mutant reverse3, and Piezo1 mutant forward3 and Piezo1 mutant reverse2. Then, the two oligos were ligated and digested using the primers forward1 and reverse2 and MluI and SalI, respectively. pSpCas9(BB)-2A-Puro (PX459) V2.0 was a gift from Feng Zhang (Addgene plasmid # 62988; http://n2t.net/addgene:62988; RRID: Addgene_62988)^22^. To construct the CRISPR-Cas9 plasmid for Piezo1-knockout (referred to as PX459-Piezo1), pSpCas9(BB)-2A-Puro (PX459) V2.0 was digested using BbsI-HF (NEB) at 37 °C for 60 min, followed by deactivation at 65 °C for 20 min. After cooling down, QuickCIP (NEB) was added and the mixture was further incubated at 37 °C for 10 min, and then deactivated at 80 °C for 2 min. The digested plasmid was purified using the Wizard SV Gel and PCR Clean-up system (Promega). A guide sequence (TATTCGAGGCCATCGTGTACCGG) to knock out human Piezo1 (accession number: NM_001142864.4) was determined using CRISPRdirect (https://crispr.dbcls.jp). Two oligos, oligo 1 and oligo 2, were phosphorylated using T4 PNK (NEB) and annealed to clone the guide sequence into the sgRNA scaffold of the plasmid. Ligation was then performed by combining the BbsI-digested PX459, the annealed oligo duplex at a 1:3 mol ratio and ligation mix (Takara Bio) at 16 °C for 30 min. The ligation mixture was introduced into *Escherichia coli* DH5α, and the insert sequence was verified by standard sequencing.

### Transfection

Transfection of plasmids was performed using Lipofectamine LTX (Invitrogen) according to the manufacturer’s protocol. Plasmids, Lipofectamine LTX, and PLUS reagent were diluted in Opti-MEM (Invitrogen) and incubated at 25 °C for 5 min for complex formation. The mixture was then added to each dish, resulting in a final plasmid concentration of 2.5 µg/mL. The culture medium was changed 4 h after the transfection. Subsequent experiments were performed 24 h after transfection.

### Establishment of Piezo1-KO A431 cell line

A431 cells (7 × 10^5^ cells/dish) were seeded onto a 60 mm dish (Iwaki) and incubated overnight. The cells were then transfected with the PX459-Piezo1 plasmid using Lipofectamine LTX (Thermo Fisher Scientific), according to the manufacturer’s instructions, after which they were washed with PBS twice at 6 h after transfection and incubated in D-MEM(+) for 1 day. Afterwards, they were washed with PBS twice and incubated in D-MEM(+) containing puromycin (1 µg/mL) (Sigma) for 3 days. Then the cells were washed twice with PBS and incubated with D-MEM(+) without puromycin for 3 days. Finally, the cells were collected and seeded onto a 96-well plate (Iwaki) by limiting dilution to isolate and culture single-cell clones.

### Sequencing of CRISPR/Cas9 target site of the Piezo1 gene

Genome DNA was extracted from A431 cells (wild type, clones #1 and #2) using GeneArt Genomic Cleavage Detection Kit (Thermo Fisher Scientific), following the manufacturer’s protocol. The sequence around the target of CRISPR/Cas9 was amplified using the following pair of primers: Piezo1 gDNA forward and Piezo1 gDNA reverse (Table S5). The amplified product was purified using Wizard SV Gel and PCR Clean-up system (Promega) and inserted into T-Vector pMD20 (Takara Bio) using DNA Ligation Kit <Mighty Mix> (Takara Bio) according to the manufacturer’s protocol. The ligation mixture was introduced into *Escherichia coli* DH5α and the insert sequence was verified by standard sequencing.

### Real-time PCR

Total RNA was extracted from A431 cells using NucleoSpin RNA Plus (Takara Bio), following the manufacturer’s protocol. The quantity and quality of RNA was measured by a nanodrop (Thermo Fisher Scientific). RNA concentration was determined by absorbance at 260 nm and RNA quality was confirmed by the 260/280 nm ratio. 2 µg of total RNA was subsequently reverse transcribed to cDNA using PrimeScript RT Master Mix (Takara Bio) according to the manufacturer’s protocol. Real-time PCR was performed using PowerUp SYBR Green Master Mix (Thermo Fisher Scientific) and 7300 Real-Time PCR System (Applied Biosystems). GAPDH was used as a reference gene.

### Dextran uptake assay

Intracellular uptake of TMR-dex70 was evaluated using confocal microscopy observation and flow cytometry analysis. A431 cells (2 × 10^5^ cells/dish and 1 × 10^5^ cells/well) were seeded onto 35 mm glass-bottomed dishes (Iwaki) and a 24-well plate (Iwaki), respectively, and incubated for 1 day. The cells were washed with PBS twice and cultured in D-MEM(−) overnight for serum-starvation. The starved A431 cells were treated with TMR-dex70 (0.5 mg/mL) and reagents as indicated on corresponding figure legends for 10 min at 37 °C. For confocal microscopy observation, the cells were then washed twice with ice-cold PBS and stained nuclei with Hoechst 33342 (5 µg/mL, Invitrogen) for 10 min. The observation was carried out using an FV1000 confocal laser scanning microscope (CLSM) system (Olympus) equipped with a 60 × objective lens (UPlanSApo, oil immersion, NA 1.35; Olympus). For flow cytometry analysis, the cells were washed twice with ice-cold PBS, detached from the plate with 0.25% trypsin in PBS for 10 min at 37 °C, added D-MEM(+) to prevent further digestion, and collected into centrifuge tubes. The cells were then centrifuged (800×*g*, 5 min, 4 °C) and the resulting pellets were washed with ice-cold PBS. The cells were centrifuged again, washed with ice-cold PBS once more and filtrated with a cell strainer. Flow cytometry analysis was performed with 10,000 gated events using an Attune NxT Flow Cytometer (Thermo Fisher Scientific). The results are shown as relative median fluorescence intensity of 10,000 counted events.

### Tfn uptake assay

A431 cells (2 × 10^5^ cells/dish and 1 × 10^5^ cells/well) were seeded onto 35 mm glass-bottomed dishes and a 24-well plate and incubated for 1 day, and then serum-starved in D-MEM(−) for 1 h prior to experiments. The cells were incubated with AF568-Tfn (20 µg/mL) in the absence or presence of Yoda1 (1.5 µM) in D-MEM(−) for 10 min at 37 °C. The cells were then acid-washed twice with Glycine–HCl buffer (with 150 mM NaCl, pH 3.0) to remove AF568-Tfn on the plasma membrane. Then the cells were fixed with 4% PFA in case of confocal microscopy observation. Microscopy observation and flow cytometry analysis of cellular uptake of Tfn were performed as described above in the method for dextran uptake assay.

### Time-lapse live cell imaging

A431 cells (2 × 10^5^ cells/dish) were seeded onto 35 mm glass-bottomed dishes (Iwaki) and incubated for 1 day. If necessary, the cells were transfected with the indicated plasmids and serum-starved prior to the experiments. The cells were washed twice with PBS, and the culture medium was replaced with D-MEM(−) (150 µL, on the glass part of the dish). The cells were placed at 37 °C in a microchamber (STXG-IX3WX-SET; Tokai Hit) attached on the FV3000 microscope stage. Reagents in D-MEM(−) (50 µL) were added to the cells to yield the final concentrations indicated in the corresponding figure legends. DIC and fluorescence images were captured every 10 or 20 s using an FV3000 confocal laser scanning microscope (CLSM) system (Olympus) equipped with a 60 × objective lens (UPlanSApo, oil immersion, NA 1.35; Olympus).

### Ca^2+^ imaging using GCaMP6s

A431 cells (2 × 10^5^ cells/dish) seeded onto 35 mm glass-bottomed dishes (Iwaki) were transfected with a plasmid to express GCaMP6s. The cells were washed with Hanks’ balanced salt solution (HBSS; 400 mg/L KCl, 60 mg/L KH_2_PO_4_, 8000 mg/L NaCl, 350 mg/L NaHCO_3_, 60 mg/L Na_2_HPO_4_·H_2_O, 1000 mg/L D-Glucose, containing 1 mM Ca^2+^, 1 mM Mg^2+^ and 20 mM HEPES at pH7.4) and the culture medium was replaced with HBSS (150 µL, on the glass part of the dish). GCaMP6s fluorescence images were acquired every 1 min as described above in the method for time-lapse live cell imaging. Fluorescence intensity was measured using ImageJ software (NIH). Yoda1-induced Ca^2+^ influx was quantified as the difference in the GCaMP6s fluorescence intensity between its maximum value (F_max_) and the basal level (F_0_).

### Ca^2+^ imaging using Fluo-8 in HEK293T expressing Piezo1 WT or mutants

HEK293T cells (2 × 10^5^ cells/dish) were seeded onto 35 mm glass-bottomed dishes and transfected with either pPiezo1(full length)-IRES2-mCherry, pPiezo1(Δ946–956)-IRES2-mCherry, pPiezo1(Δ944–946)-IRES2-mCherry or empty vector (pIRES2-mCherry). HEK293T cells expressing mCherry were considered to express Piezo1 WT or the mutants. The cells were treated with Fluo-8 AM (5 µM, AAT Bioquest) for 45 min and then washed twice with PBS. The cells were then washed with Hanks’ balanced salt solution (HBSS; 400 mg/L KCl, 60 mg/L KH_2_PO_4_, 8000 mg/L NaCl, 350 mg/L NaHCO_3_, 60 mg/L Na_2_HPO_4_·H_2_O, 1000 mg/L D-Glucose, containing 1 mM Ca^2+^, 1 mM Mg^2+^ and 20 mM HEPES at pH 7.4) and the culture medium was replaced with HBSS (150 µL, on the glass part of the dish). Fluo-8 fluorescence images were acquired every 1 min as described above in the method for time-lapse live cell imaging. Fluorescence intensity was measured using ImageJ software (NIH). Yoda1-induced Ca^2+^ influx was quantified as the difference in the Fluo-8 fluorescence intensity between its maximum value (F_max_) and the basal level (F_0_).

### Membrane ruffling assay

A431 cells (2.5 × 10^5^ cells/dish) were seeded onto 35 mm glass-bottomed dishes, cultured for 1 day and then serum-starved overnight prior to experiments. The cells were treated with reagents for 5 min at 37 °C, fixed with 4% paraformaldehyde in PBS for 10 min, and permeabilized with 0.1% Triton X-100 in PBS for 4 min. The cells were then incubated with 1% BSA in PBS for 30 min to block non-specific binding prior to staining F-actin with rhodamine-conjugated phalloidin (Invitrogen) for 30 min. The observations were carried out using the FV1000 CLSM system (Olympus) equipped with a 40 × objective lens (UPlanSApo, NA 0.95; Olympus). Among the cells at the margin of the colony, the cells with over 12 µm accumulation of phalloidin were counted as cells with ruffles.

### Scanning electron microscopy

A431 cells were stimulated with EGF (20 nM) in the absence or presence of Yoda1 (1.5 µM) for 5 min and then fixed with 2% glutaraldehyde and 4% paraformaldehyde in 0.1 M cacodylate buffer (pH 7.4) for 2 h, washed with cacodylate buffer, and post-fixed with 1% osmium tetroxide in cacodylate buffer for 2 h. After washing with distilled water, the specimens were subjected to the conductive staining with 1% buffered osmium tetroxide and 1% tannic acid (O-T-O methods). The specimens were then dehydrated in a graded series of ethanol and critical-point drying (Hitachi, Ltd. Critical Point Dryer HCP-1), coated with a thin layer (3 nm) of osmium coater (Vacuum Device Inc.; HPC-30W), and then observed with a field-emission scanning electron microscope (Hitachi S-4800, Tokyo, Japan) at 2 kV acceleration voltage.

### Rac1 activation assay

The Rac1 activation assay was conducted using the Rac1 Pull-Down Activation Assay Biochem Kit (Cytoskeleton) according to the manufacturer’s protocol. A431 cells (9 × 10^5^ cells/dish) were seeded onto a 100 mm dishes (Iwaki), cultured for 2 days and serum-starved overnight prior to experiments. The cells were treated with reagents for 3 min at 37 °C, lysed with 240 µL of ice-cold lysis buffer [50 mM Tris–HCl (pH 7.5), 10 mM MgCl_2_, 0.5 M NaCl, 2% IGEPAL]. The lysates were cleared by centrifugation at 10,000×*g* for 1 min at 4 °C. 30 µL of the lysate supernatant was used for protein concentration measurement and preparation of total cell lysate for detecting total Rac1. The remaining supernatant was incubated for 1 h at 4 °C with GST-tagged PAK-PBD beads. After washing the beads, bound proteins were eluted with SDS-loading buffer. The lysates were applied into a polyacrylamide gel (SuperSep Ace, 5–20%, 13 well; FUJIFILM Wako Pure Chemical Corporation) and fractionated by SDS-PAGE. The blots were transferred to a polyvinylidene difluoride membrane using Trans-Blot Turbo Transfer System (Bio-Rad), blocked with 5% skim milk in TBS containing 0.05% Tween-20 (TBST) for 1 h at 25 °C, and then incubated overnight at 4 °C with appropriate primary antibodies in 3% skim milk in TBST. After washing the membrane with TBST for 10 min three times, the blots were further incubated with appropriate horseradish peroxidase (HRP)-conjugated secondary antibodies in 3% skim milk in TBST for 1 h at 25 °C. After washing the membrane with TBST for 10 min three times, chemiluminescence was detected using ECL prime (GE Healthcare) and LAS3000 mini (FUJIFILM). The images were analyzed using ImageJ software (NIH).

### Western blot

A431 cells (3 × 10^5^ cells/well) were seeded on a 6-well plate (Iwaki), cultured for 1 day and serum-starved overnight prior to experiments. The cells were treated with EGF in the presence or absence of Yoda1 for 5 min at 37 °C, lysed with ice-cold RIPA buffer [50 mM Tris–HCl (pH 7.6), 150 mM NaCl, 1 mM EDTA, 1% Triton X-100, 0.1% SDS, 0.1% sodium deoxycholate] containing protease inhibitor cocktails (Roche) and phosphatase inhibitor cocktails (Roche), and lysates were cleared by centrifugation at 16,000×*g* for 20 min at 4 °C. The protein concentrations of the lysates were measured by BCA protein assay using Pierce BCA Protein Assay Kit (Thermo Fisher Scientific) and then unified to 0.5 µg/µL. SDS-PAGE, antibody treatment and detection were performed described as above in the method for Rac1 activation assay using 5% BSA in TBST as blocking solution. When detecting EGFR and Vav2, the membrane was subjected for the first immunoblots (pEGFR or pVav2), stripped by immerging the membrane in Restore PLUS Western Blot Stripping Buffer (Thermo Fischer Scientific) for 5 min and washed twice with TBST for 10 min and then subjected to the second immunoblots (EGFR or Vav2).

### Intracellular pH measurement

A431 (2.5 × 10^5^ cells/dish) cells were seeded on 35 mm glass-bottomed dishes (Iwaki) and incubated for 1 day. The cells were incubated with SNARF-5F AM (20 µM, Invitrogen) in D-MEM(−) for 30 min at 37 °C, washed twice with PBS, and then incubated with Yoda1 (1.5 µM) in D-MEM(−) for 10 min at 37 °C. EIPA (25 µM, 30 min, 37 °C) was used as a positive control which decreases intracellular pH. The observation was done using the FV1000 CLSM system (Olympus) equipped with a 40 × objective lens (UPlanSApo, NA 0.95; Olympus). SNARF-5F AM was excited at 559 nm and images were acquired in the range of 570–600 nm and 630–660 nm to evaluate emission signal at 585 nm and 645 nm, respectively. Fluorescence intensity was measured using ImageJ software (NIH) and ratio of emission signal of 585/645 nm was calculated. For establishing calibration curve, cells were incubated with SNARF-5F AM as above, washed twice with calibration buffer (130 mM KCl, 10 mM NaCl, 1 mM MgSO_4_, 10 mM MOPS) at pH 6.2, 6.6, 7.0, 7.4, 7.8, or 8.2, and then incubated with calibration buffer containing 10 µg/mL nigericin for 15 min at 25 °C to equilibrate the intracellular pH with extracellular pH.

### WST-8 assay

The WST-8 assay was performed using the Cell Counting Kit-8 (Dojindo), according to the manufacturer’s protocol. A431 cells (1 × 10^4^ cells/well) were seeded onto a 96-well plate (Iwaki) and incubated for 1 day. The cells were treated with DMSO or Yoda1 (1.5 and 3 µM) in D-MEM(+) for 4 h at 37 °C. In case of starved condition, the cells were starved overnight and then treated with DMSO or Yoda1 (1.5 and 3 µM) in D-MEM(−) for 30 min or 4 h at 37 °C. The cells were then washed twice with PBS before adding 100 µL D-MEM(−) and 10 µL of WST-8 reagent to each well. The cells were further incubated for 2 h at 37 °C followed by measuring the absorbance at 450 nm.

### Statistical analysis

All data are presented as the mean ± standard error of the mean (s.e.m.) of three independent biological replicates (n = 3) unless otherwise specified. All statistical analyses were performed using JMP Pro 14 (SAS Institute Inc.). For comparison of two groups, an unpaired Student’s *t* test was used. For multiple comparison analyses, one-way analysis of variance (ANOVA) followed by Tukey–Kramer’s post hoc test or Dunnett’s post hoc test was used. The calculated p-values were considered significant at p < 0.05.

## Supplementary Information

Supplementary Information.

Supplementary Movie 1.

Supplementary Movie 2.

Supplementary Movie 3.

Supplementary Movie 4.
