# Publisher Correction: Piezo1 activation using Yoda1 inhibits macropinocytosis in A431 human epidermoid carcinoma cells

**DOI:** 10.1038/s41598-022-12161-0

**Published:** 2022-05-12

**Authors:** Masashi Kuriyama, Hisaaki Hirose, Toshihiro Masuda, Masachika Shudou, Jan Vincent V. Arafiles, Miki Imanishi, Masashi Maekawa, Yuji Hara, Shiroh Futaki

**Affiliations:** 1 Institute for Chemical Research, Kyoto University, Uji, Kyoto 611-0011 Japan; 2 Division of Analytical Bio-Medicine, Advanced Research Support Center, Ehime University, Toon, Ehime 791-0295 Japan; 3 Division of Cell Growth and Tumor Regulation, Proteo-Science Center, Ehime University, Toon, Ehime 791-0295 Japan; 4 Department of Biochemistry and Molecular Genetics, Ehime University Graduate School of Medicine, Toon, Ehime 791-0295 Japan; 5 Department of Synthetic Chemistry and Biological Chemistry, Graduate School of Engineering, Kyoto University, Katsura, Kyoto 615-8510 Japan; 6 Present Address: Division of Physiological Chemistry and Metabolism, Graduate School of Pharmaceutical Sciences, Keio University, Minato-ku, Tokyo, 105-8512 Japan; 7 Present Address: School of Pharmaceutical Sciences, University of Shizuoka, Shizuoka, 422-8526 Japan

Correction to: *Scientific Reports* https://doi.org/10.1038/s41598-022-10153-8, published online 15 April 2022

The original version of this Article contained an error in the spelling of the author Jan Vincent V. Arafiles which was incorrectly given as Jan Vincent Arafiles.

The original Article has been corrected.

