# Supplementary material for: Piezo1 activation using Yoda1 inhibits macropinocytosis in A431 human epidermoid carcinoma cells
Source: Sci Rep. 2022 Apr 15;12:6322. doi: 10.1038/s41598-022-10153-8 (PMC9012786; doi:10.1038/s41598-022-10153-8)
Supplement: Supplementary file 1 — Supplementary Information. [file 41598_2022_10153_MOESM1_ESM.docx]

**Piezo1 activation using Yoda1 inhibits macropinocytosis in A431 human epidermoid carcinoma cells**

Masashi Kuriyama^1,#^, Hisaaki Hirose^1,#,^*, Toshihiro Masuda^1^, Masachika Shudou^2^, Jan Vincent V. Arafiles^1^, Miki Imanishi^1^, Masashi Maekawa^3,4,5^, Yuji Hara^6,7^, and Shiroh Futaki^1,^*

**Supplementary Information**

**
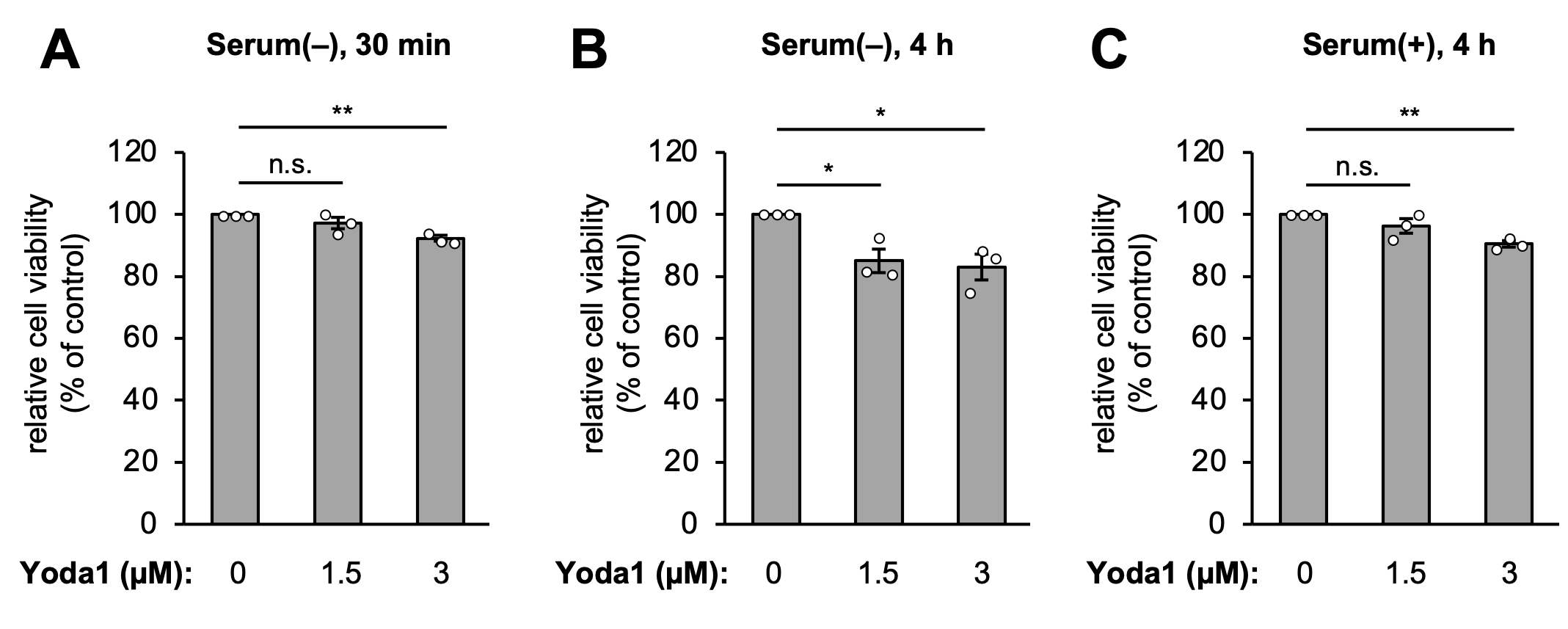
**

**Supplementary Figure S1.** The cell viability of A431 cells treated with Yoda1 at the indicated concentration. (A) The starved A431 cells were treated with Yoda1 for (A) 30 min and (B) 4 h in in D-MEM(–). (C) A431 cells were treated with Yoda1 for 4 h in D-MEM(+). **, p < 0.01; *, p < 0.05; n.s., not significant (one-way ANOVA followed by Dunnett’s post hoc test).

**
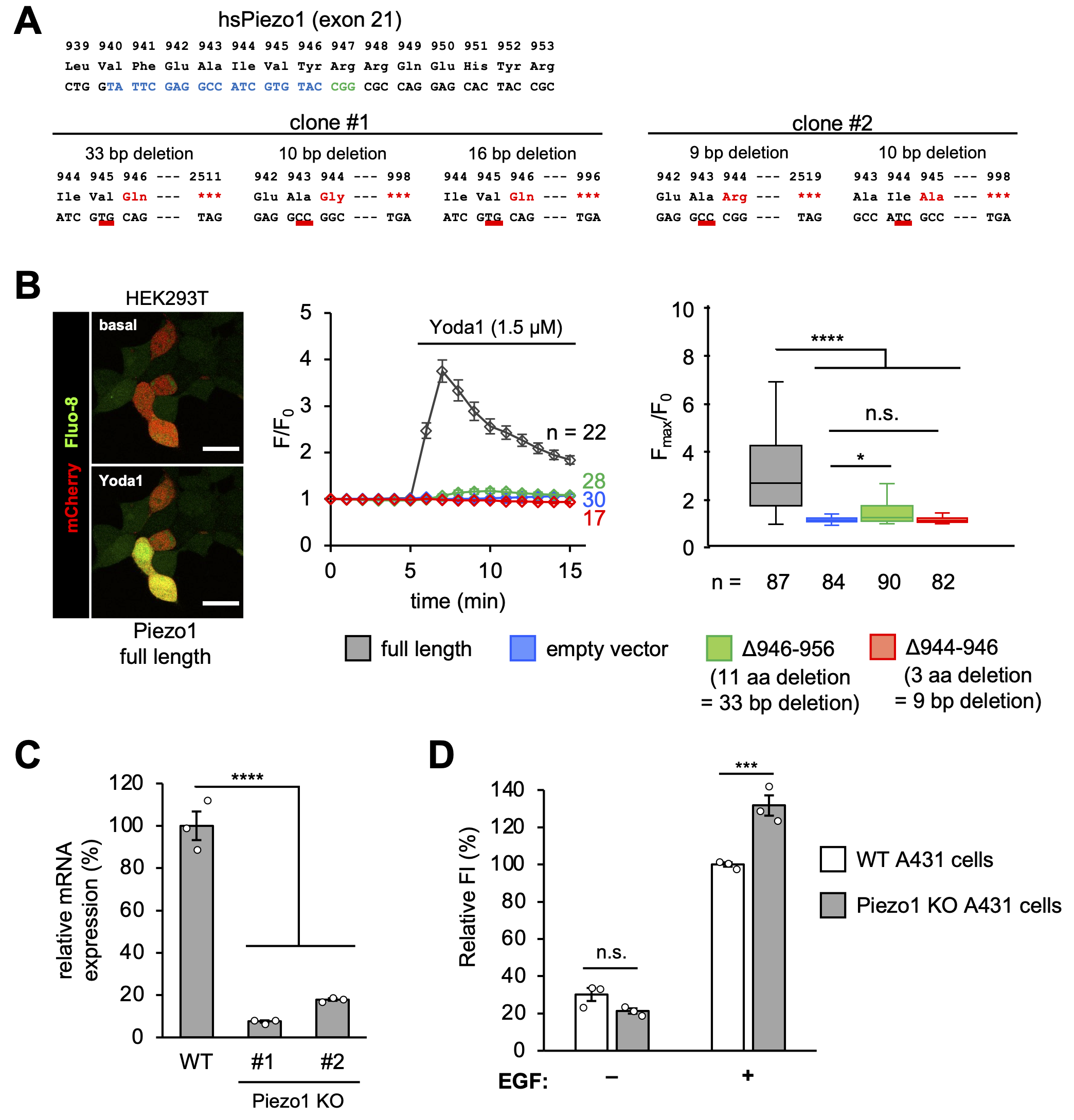
**

**Supplementary Figure S2.** Supporting data for characterization of Piezo1 knockout A431 cells. (**A**) CRISPR/Cas9 target site of the Piezo1 gene. The guide sequence and the protospacer adjacent motif are showed in blue and green characters, respectively. CRISPR/Cas9-mediated deletions are showed in red characters. (**B**) HEK293T cells were transfected with indicated vector, loaded with Fluo-8 AM (5 µM) and then Fluo-8 fluorescence intensity was recorded every 1 min. Transient gene expression was confirmed by IRES2 dependent co-expression of mCherry. Yoda1 (1.5 µM) was added 5 min after start of time-lapse imaging. Left: Representative images before and after Yoda1 addition. Middle: Representative time-course of relative fluorescence intensity of Fluo-8 AM. Data represent the mean ± s.e.m.. Right: Quantification of maximum Yoda1-induced GCaMP6s intensity increase. (n ≥ 82 cells for each condition pooled from three independent experiments.) Box and whiskers graph: line, median; box, upper and lower quartiles; whiskers, maxima and minima; circle plots, outliers. Scale bars, 25 µm. ****, P < 0.0001; *, P < 0.05; n.s., not signficant (one-way ANOVA followed by Tukey–Kramer’s post hoc test). (**C**) Expression of Piezo1 in A431 cells (WT, KO clone #1, 2). Piezo1 mRNA expression level was determined by qPCR. The GAPDH shows relative mRNA expression level of Piezo1 in each condition. Data represent mean ± s.d. of triplicate samples from one of two independent experiments. (**D**) Flow cytometry analysis of EGF-stimulated TMR-dex70 uptake for 10 min in WT or Piezo1-KO A431 cells. *, p < 0.05; ***, p < 0.001; ****, p < 0.0001; n.s., not significant (one-way ANOVA followed by Tukey–Kramer’s post hoc test (B, D) or one-way ANOVA followed by Dunnett’s post hoc test (C))


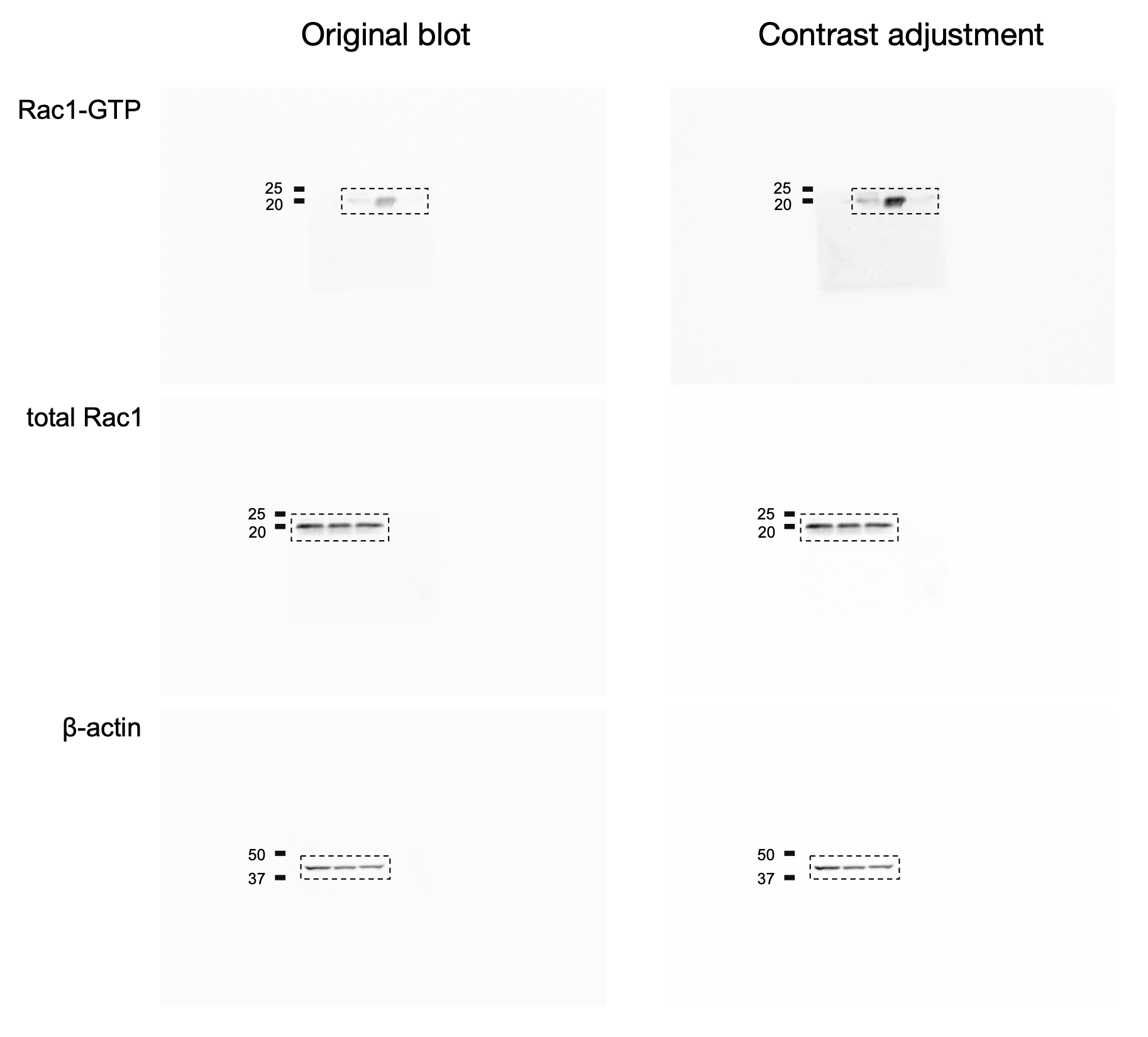


**Supplementary Figure S3.** The uncropped data of Rac1 pull-down assay (Left, original blot; Right, adjusted contrast). The dotted line indicates the section shown in the main figure (Fig. 3). The black bands show molecular weight markers (kDa).

**
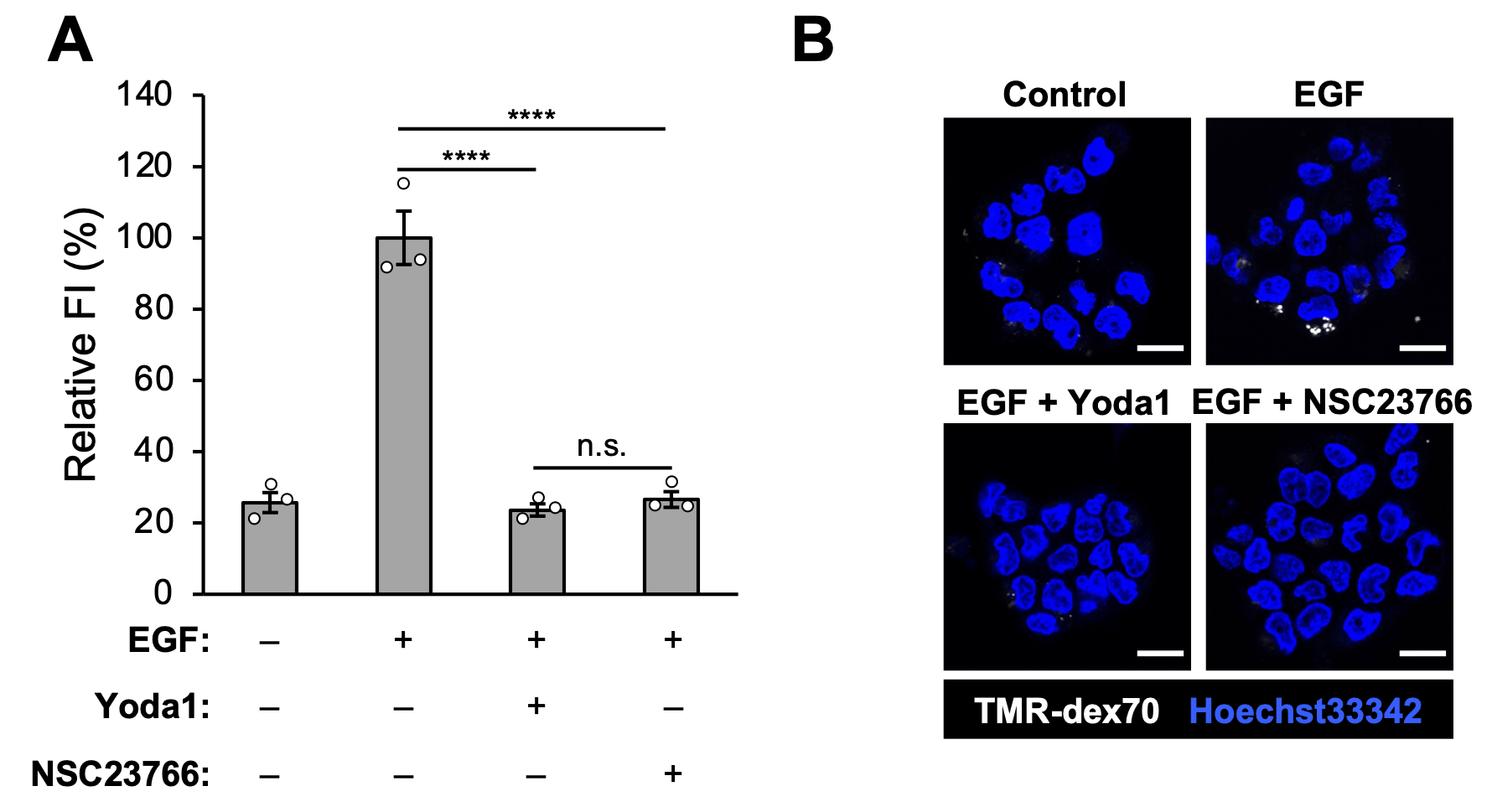
**

**Supplementary Figure S4.** (A) Flow cytometry analysis of EGF-stimulated TMR-dex70 uptake for 10 min in the presence of Yoda1 (1.5 µM) or the Rac1 inhibitor, NSC23766 (100 µM). ****, p < 0.0001; n.s., not significant (one-way ANOVA followed by Tukey-Kramer’s post hoc test). (B) Confocal microscopy observation of TMR-dex70 uptake in A431 cells treated as described in (A).

**
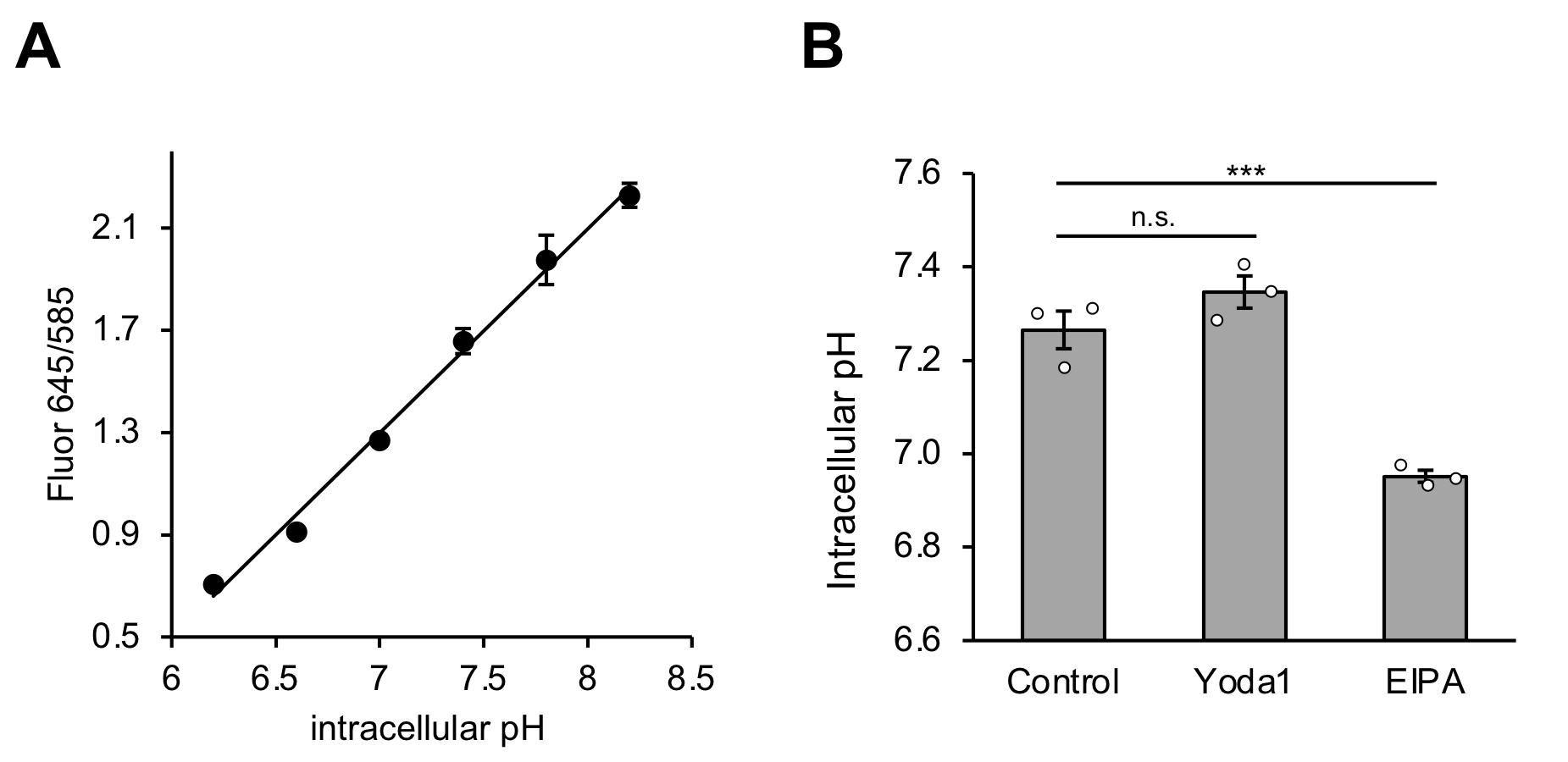
**

**Supplementary Figure S5.** Yoda1 does not change intracellular pH. (**A**) Relationship between fluorescence intensity ratio (645/585 nm) of SNARF-5F AM and intracellular pH. Fluorescence intensity ratio (645/585 nm) of SNARF-5F AM was plotted against respective pH and linear regression analysis was performed to obtain the calibration curve. Data represent mean ± s.e.m. (n = 150 cells for each condition from three independent experiments). (**B**) Intracellular pH measurement using SNARF-5F AM in starved A431 cells treated either with Yoda1 (1.5 µM) for 10 min or EIPA (25 µM) for 30 min in D-MEM(–). EIPA was used as a positive control to reduce intracellular pH. The intracellular pH value was obtained from the calibration curve in (A). Data represent mean ± s.e.m. (n = 150 cells for each condition from three independent experiments). ***, p < 0.001; n.s., not significant (one-way ANOVA followed by Dunnett’s post hoc test).


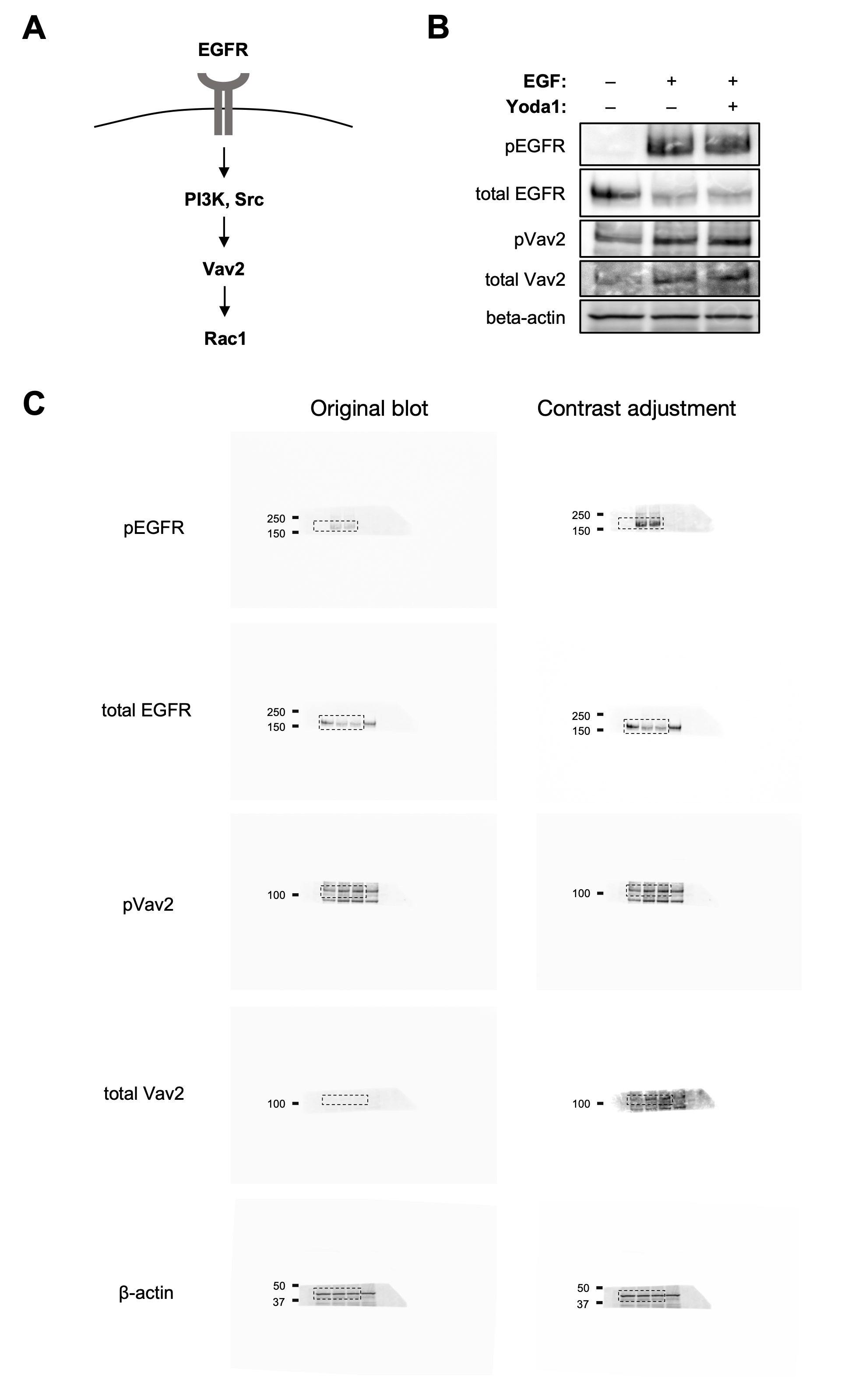


**Supplementary Figure S6.** Yoda1 does not inhibit phosphorylation of EGFR and Vav2. (**A**) Schematic diagram showing the pathway of Rac1 activation by EGF. (**B**) Western blot analysis. A431 cells were treated with EGF (20 nM) and Yoda1 (1.5 μM) for 5 min, and total cell lysates were analyzed by performing SDS-PAGE followed by western blot. The experiments were performed at least twice with similar results. (C) The uncropped data of Western blot analysis (Left, original blot; Right, adjusted contrast). The dotted line indicates the section shown in B. The black bands show molecular weight markers (kDa).


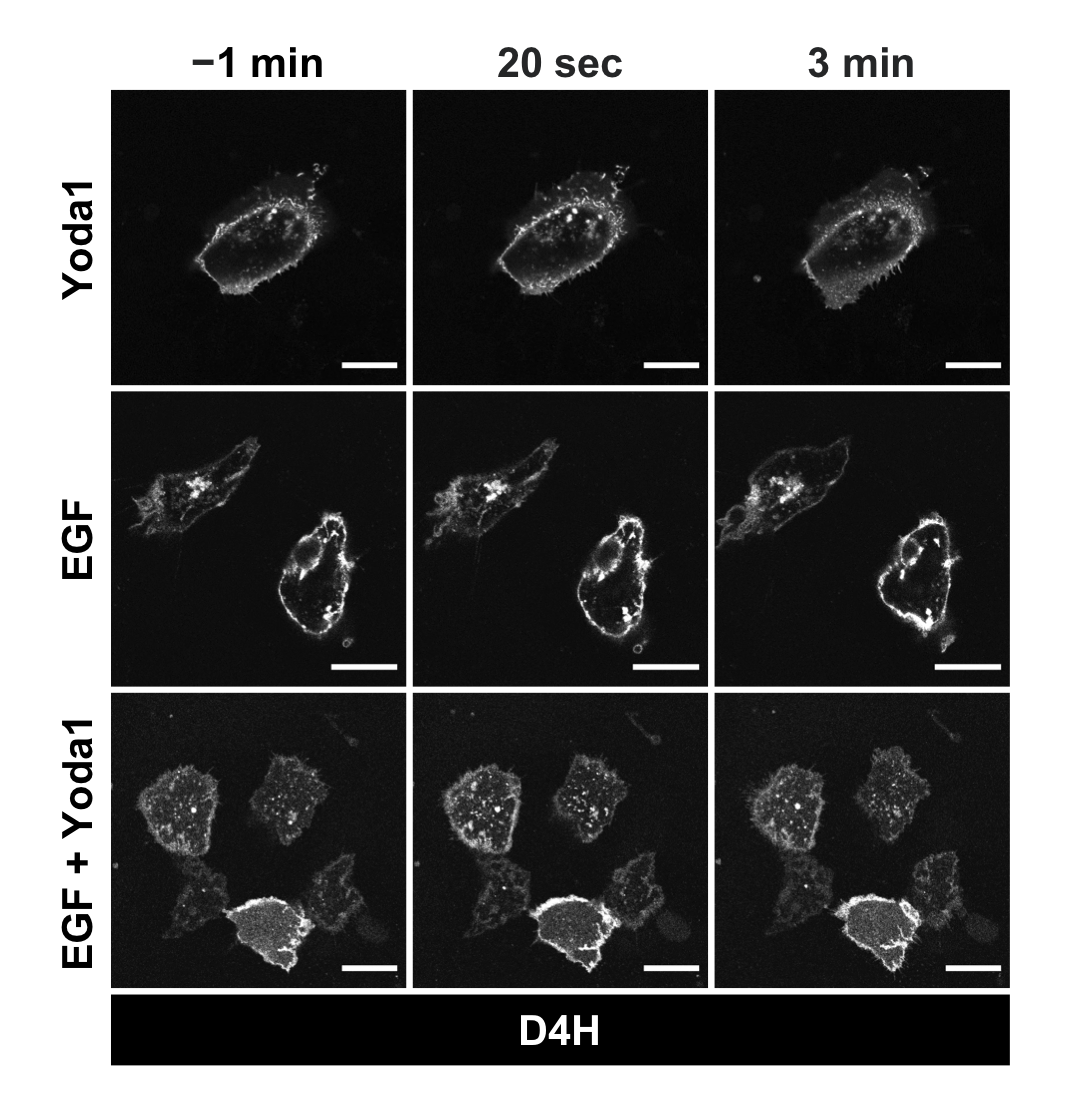


**Supplementary Figure S7.** Yoda1 does not affect cholesterol localization. A431 cells were transfected with mCherry-tagged D4H, a specific probe for cholesterol, serum-starved for 4 h, and then mCherry-D4H fluorescence images were acquired every 20 sec. The time of EGF (20 nM) and Yoda1 (1.5 µM) addition was defined as time = 0. Images at indicated time points (1 min before adding reagents and 20 sec, 3 min after addition) are shown. Scale bars, 20 µm.


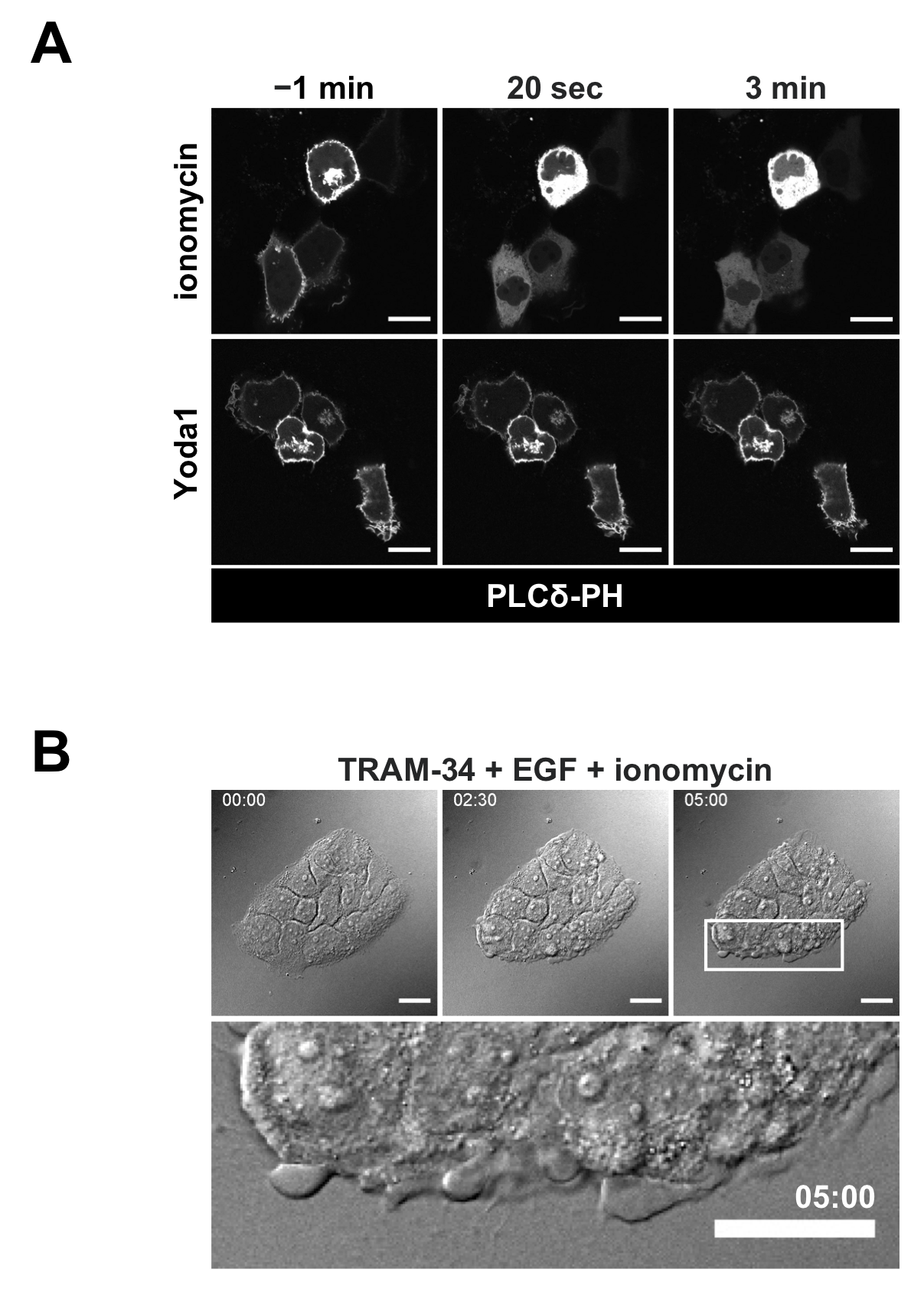


**Supplementary Figure S8.** Yoda1 does not induce PI(4,5)P2 depletion. (A) A431 cells were transfected with GFP-tagged PLCδ-PH, a specific probe for PI(4,5)P2, and then GFP-PLC-PH fluorescence images were acquired every 20 s. The time of ionomycin (5 µM) or Yoda1 (1.5 µM) addition was defined as time = 0. Images at indicated time points (1 min before adding reagents, 20 s and 3 min after addition) are shown. (B) Live imaging shows that ionomycin inhibits EGF-induced ruffle also in the presence of TRAM-34. A431 cells were pretreated with a KCa3.1 inhibitor TRAM-34 (10 µM) for 5 min and then stimulated with EGF (20 nM) in the presence of ionomycin (5 µM). Time-lapse imaging was started immediately after adding EGF and ionomycin. DIC images at the indicated time points (0 min, 2.5 min and 5 min) are shown. The bottom image shows an enlarged view of the area outlined by the white square in the image at 5 min. Scale bars, (A, B) 20 µm.

| Table S1 |  |  |  |
| --- | --- | --- | --- |
| **Reagent name** | **Source** | **Catalog #** | **Additional information** |
| TMR-dextran 70 kDa | Thermo Fischer | D1819 |  |
| Alexa Fluor 568-Transferrin | Thermo Fischer | T23365 |  |
| rhodamine phalloidin | Thermo Fischer | R415 |  |
| recombinant EGF | Sigma-Aldrich | SRP3027 |  |
| Yoda1 | Sigma-Aldrich | SML1558 | Piezo1 agonist |
| EIPA | Sigma-Aldrich | A3085 | macropinocytosis inhibitor |
| TRAM-34 | Tocris | 2946 | KCa3.1 inhibitor |
| SKA-31 | Tocris | 3670 | KCa3.1 agonist |
| ionomycin | AdipoGen | AG-CN2-0416 | Ca2+ ionophore |

**Supplementary Table S1.** Reagents used in this study.

| Table S2 |  |  |  |
| --- | --- | --- | --- |
| **Antibody name** | **Source** | **Catalog #** | **Dilution** |
| anti-Rac1 | Merck Millipore | 05-389 | 1:1000 |
| anti-EGFR | Cell Signaling Technology | 4267 | 1:1000 |
| anti-pEGFR (Y1068) | Cell Signaling Technology | 2234 | 1:1000 |
| ant-Vav2 | Cell Signaling Technology | 2848 | 1:1000 |
| anti-pVav2 (Y172) | Abcam | ab86695 | 1:1000 |
| anti-rabbit IgG (HRP) | Cell Signaling Technology | 7074 | 1:2000 |
| anti-mouse IgG (HRP) | GeneTex | GTX213111-01 | 1:5000 |
| anti-β-actin | Sigma-Aldrich | A2228 | 1:10000 |

**Supplementary Table S2.** Antibodies used in this study.

| Table S3 |  |  |  |
| --- | --- | --- | --- |
| **Cell line name** | **Source** | **Catalog #** | **Additional information** |
| A431 | ATCC | CRL-1555 | epidermoid carcinoma cell line |
| HEK293T | RIKEN BRC | RCB2202 | embryonic kidney cell line |

**Supplementary Table S3.** Cell lines used in this study.

| Table S4 |  |  |
| --- | --- | --- |
| **Plasmid or cDNA name** | **Source** | **Catalog #** |
| human Piezo1 cDNA | Promega | FXC01061 |
| pGP-CMV-GCaMP6s | This study (materials and methods) |  |
| pIRES2-mCherry | This study (materials and methods) |  |
| pPiezo1-IRES2-mCherry | This study (materials and methods) |  |
| pPiezo1(Δ946-956)-IRES2-mCherry | This study (materials and methods) |  |
| pPiezo1(Δ944-946)-IRES2-mCherry | This study (materials and methods) |  |
| pSpCas9(BB)-2A-Puro (PX459) V2.0 | Addgene | 62988 |
| pGFP-C1-PLCδ-PH | DOI: 10.1242/jcs.164715 |  |
| pmCherry-C1-D4H | DOI: 10.1242/jcs.164715 |  |

**Supplementary Table S4.** Plasmids used in this study.

| Table S5 |  |
| --- | --- |
| **Primer name** | **Sequence** |
| Piezo1 mutant forward1 | 5´-CCGGCTTCTCGGACG-3´ |
| Piezo1 mutant forward2 | 5´-CGAGGCCATCGTGCAGCTGGCCCCGCTG-3´ |
| Piezo1 mutant forward3 | 5´-GTATTCGAGGCCCGGCGCCAGGAGCAC-3´ |
| Piezo1 mutant reverse1 | 5´-CGGGGCCAGCTGCACGATGGCCTCGAATAC-3´ |
| Piezo1 mutant reverse2 | 5´-CCAAAGGCCCAGAAGC-3´ |
| Piezo1 mutant reverse3 | 5´-CTCCTGGCGCCGGGCCTCGAATACCAGC-3´ |
| Piezo1 gDNA forward | 5´-CCAACAGCACCAACTTGCTG-3´ |
| Piezo1 gDNA reverse | 5´-AGGAGTCCAGGGAAGCCGT-3´ |
| Piezo1 forward | 5´-AGGCGCATCAGTCTACGTTT-3´ |
| Piezo1 reverse | 5´-GCTTGGCCTCTTCTCTCTCC-3´ |
| GAPDH forward | 5´-CCTGTTCGACAGTCAGCCG-3´ |
| GAPDH reverse | 5´-CGACCAAATCCGTTGACTCC-3´ |
| oligo 1 | 5´-CACCGTATTCGAGGCCATCGTGTAC-3´ |
| oligo 2 | 5´-AAACGTACACGATGGCCTCGAATAC-3´ |

**Supplementary Table S5.** Primers used in this study.

**Movie description**

**Movie 1.** EGF induces peripheral ruffle formation. Differential interference contrast (DIC) movie of A431 cells treated with EGF (20 nM). Time-lapse imaging was started immediately after adding EGF, and frames were acquired every 10 s. Video was sped up 100× (ten frames per second) over real time. Scale bar, 20 μm.

**Movie 2.** Yoda1 inhibits ruffle peripheral formation. Differential interference contrast (DIC) movie of A431 cells treated with EGF (20 nM) and Yoda1 (1.5 μM). Time-lapse imaging was started immediately after adding EGF and Yoda1, and frames were acquired every 10 s. Video was sped up 100× (ten frames per second) over real time. Scale bar, 20 μm.

**Movie 3.** TRAM-34 recovers peripheral formation in the presence of Yoda1. Differential interference contrast (DIC) movie of A431 cells pretreated with TRAM-34 (10 μM) for 5 min and then treated with EGF (20 nM) and Yoda1 (1.5 μM). Time-lapse imaging was started immediately after adding EGF and Yoda1, and frames were acquired every 10 s. Video was sped up 100× (ten frames per second) over real time. Scale bar, 20 μm.

**Movie 4.** TRAM-34 does not recover peripheral formation in the presence of ionomycin. Differential interference contrast (DIC) movie of A431 cells pretreated with TRAM-34 (10 μM) for 5 min and then treated with EGF (20 nM) and ionomycin (5 μM). Time-lapse imaging was started immediately after adding EGF and ionomycin, and frames were acquired every 10 s. Video was sped up 100× (ten frames per second) over real time. Scale bar, 20 μm.
